# Adamantane-thiazole hybrids and related derivatives: synthesis, crystal structures,* in vitro* antibacterial, antifungal, and anti-proliferative activities

**DOI:** 10.1186/s13065-025-01706-9

**Published:** 2026-01-20

**Authors:** Heidi S. Abd El-Monaem, Mahmoud B. El-Ashmawy, Naglaa I. Abdel-Aziz, Olivier Blacque, E. Habib, Subbiah Thamotharan, Ali A. El-Emam

**Affiliations:** 1 Department of Medicinal Chemistry, Faculty of Pharmacy, Mansoura University, Mansoura, 35516 Egypt; 2 Department of Chemistry, University of Zurich, Winterthurerstrasse 190, 8057 Zurich, Switzerland; 3 Department of Microbiology and Immunology, Faculty of Pharmacy, Mansoura University, Mansoura, 35516 Egypt; 4 Biomolecular Crystallography Laboratory and DBT-Bioinformatics Center, School of Chemical and Biotechnology, SASTRA Deemed University, Thanjavur, 613 401 India

**Keywords:** Adamantane, Thiazole, Antimicrobial activity, Anti-proliferative activity, Single crystal XRD, Molecular docking

## Abstract

Series of 2-(adamantan-2-ylidene)-*N*-substituted hydrazine-1-carbothioamide derivatives **5a-d** and (*E*)-2-[(adamantan-2-ylidene)hydrazono]-3,4-diaryl-2,3-dihydrothiazole derivatives **7a-l** were prepared and their structures were confirmed. In vitro antimicrobial evaluation of compounds **5a-d** and **7a-l** against different pathogenic bacterial and fungal strains revealed that compounds **5a**, **5b**, **5c**, **7a**, **7f**, **7i** and **7k** displayed notable effectiveness against the Gram-positive bacteria, *Staphylococcus aureus* and *Bacillus subtilis*, and the Gram-negative *Escherichia coli*, and all compounds lacked antifungal activity. In addition, compounds **5a**, **5b**, **5c**, **7f** and **7 L** displayed marked anti-proliferative activity particularly against HepG-2 and MCF-7 cancer cell lines (IC_50_ < 25 µM). The structures of compounds **5c**, **7a** and **7f** were confirmed by single-crystal X-ray diffraction studies. Compound **5c** crystallized with a lattice water molecule and is stabilized by N–H···Cl/S, and C–H···O/Cl/π interactions. The crystal packing features of **7a** and **7f** are very similar, despite the presence of a methoxy substituent in **7f**. Compound **7a** is stabilized by C–H···N/S/π interactions and a chalcogen bond (S···π), whereas compound **7f** is stabilized by C–H···N/S/π interactions as well as C–H···O interactions involving the methoxy oxygen as an acceptor. Molecular docking studies of the most potent antibacterial adamantane-thiazole derivatives **7f** and **7i** showed good affinity towards dehydrosqualene synthase (*Sa*CrtM) from *Staphylococcus aureus*. Meanwhile, potent anti-proliferative compounds **5a**, **5b**, and **7f** showed marked affinity the urokinase-type plasminogen activator receptor (uPAR).

**Supplementary Information:**

The online version contains supplementary material available at 10.1186/s13065-025-01706-9.

## Introduction

**Fig. 1:**
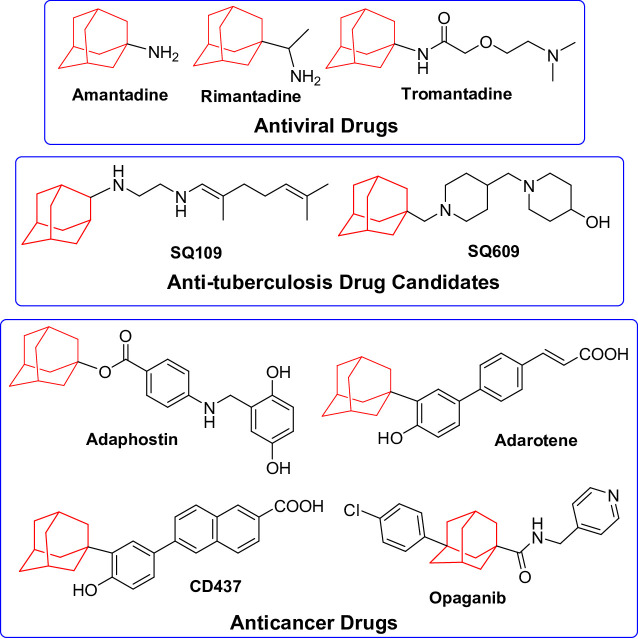
Examples of adamantane-based chemotherapeutic agents

Microbial infection and cancer represent substantial global health challenges. Antimicrobial therapy, while effective, is confronted by numerous limitations, including high cost, adverse side effects, and the rising prevalence of antimicrobial resistance [1]. Resistance often results from genetic mutations and gene recombination, further exacerbated by the excessive and improper use of antibiotics [2]. Cancer, characterized by uncontrolled cell proliferation, and metastasis, accounts for substantial morbidity and mortality of 16.8% of global deaths [3]. Both antimicrobial resistance and cancer difficulties have rendered the development of new chemotherapeutic agents a primary focus in medicinal chemistry research, aiming to identify potent molecules with enhanced specificity and diminished toxicity compared to current alternatives. Adamantane derivatives have gained considerable attention in medicinal chemistry owing to their distinctive structural and biological characteristics [4, 5]. The lipophilic cage structure of adamantane enhances membrane permeability and improves metabolic stability [6]. The potential of using adamantane-containing compounds as therapeutic agents began with the discovery that amantadine [7] and rimantadine [8] that effectively addressed Influenza A infections. Subsequently, tromantadine [9] emerged as a powerful antiviral intervention for skin conditions caused by herpes simplex virus. The ethylenediamine-linked adamantane analogue SQ109 [10] was further developed as potent drug that inhibits multidrug-resistant tuberculosis *via* targeting mycolic acid transport [11]. The related dipiperidine derivative SQ609 [12] was also approved as efficient therapies against drug-susceptible and drug-resistant *M. tuberculosis* strains, it reduces intracellular *M. tuberculosis* growth by over 90%. Beyond antiviral and anti-tuberculosis applications, some adamantane-containing compounds displayed significant promises in anticancer therapy. Adaphostin [13], an adamantyl ester, inhibits protein tyrosine kinase in chronic myelogenous leukemia. Adarotene (ST1926) is an adamantyl retinoid that displayed notable anti-growth effects in various cancers, including leukemia, prostate cancer, and non-small cell lung cancer [14]. The related adamantane retinoid CD437 [15], has shown promise as an anticancer drug by disrupting DNA polymerase. Later, Opaganib [16] was introduced as a powerful sphingosine kinase inhibitor, and received approval for treating advanced solid tumors. Opaganib has also appeared as a potential therapy for severe pneumonia associated with COVID-19 [17] (Fig. 1).

**Fig. 2:**
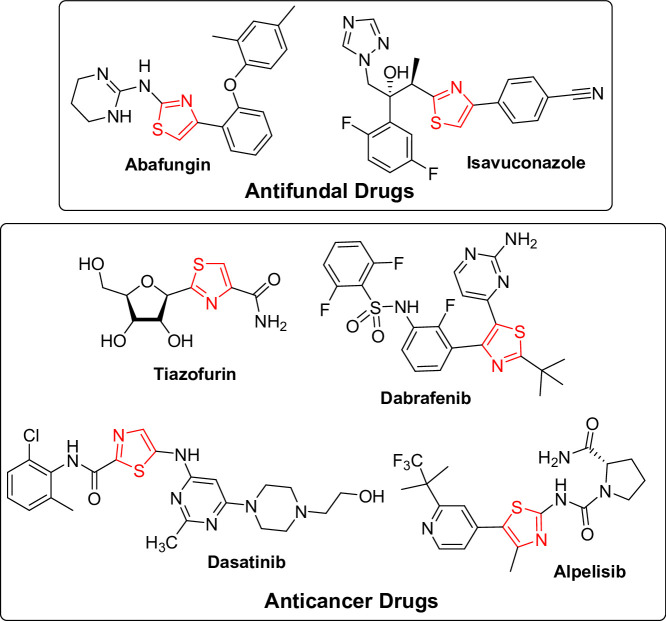
Examples of thiazole-based chemotherapeutic agents

In parallel, thiazole heterocycle was recognized as a vital building block in the creation of an array of bioactive drugs [18, 19]. Compounds featuring thiazole ring have demonstrated utility as chemotherapeutic agents, including antibacterial [20, 21], antiviral [22], and anticancer [23–26] activities. In all classical penicillins, the essential β-lactam ring is fused with thiazole. On the other hand, the medical significance of thiazole-containing compounds was illustrated by the broad-spectrum antifungal abafungin that directly impairs the fungal cell membrane, for the treatment of dermatomycoses [27]. Isavuconazole [28] has also been approved for the treatment of invasive aspergillosis and mucormycosis. As regards the anticancer activity, there are several thiazole derivatives that exhibit significant selectivity and binding affinity for pivotal molecular targets implicated in cancer progression. Tiazofurin [29], a thiazole nucleoside analogue that inhibits inosine monophosphate dehydrogenase, has displayed promise in treating hematologic cancers. Dabrafenib [30] acts as an inhibitor of the associated enzyme B-Raf, which plays a role in the regulation of cell growth, and is used in melanoma and lung cancer. Dasatinib [31] is a multi-kinase inhibitor for Philadelphia chromosome-positive leukemia. Alpelisib [32] is a PI3Kα-selective inhibitor, that has been found effective in breast cancer management (Fig. 2).

On the other hand, molecular hybridization has become a common strategy in the drug design, seeking to improve target affinity, therapeutic efficacy, and pharmacological profile relative to parent molecules [33]. This method involves the merging of two or more pharmacologically active entities within a single chemical structure, resulting in molecule that can influence different biological targets or mitigate adverse effects commonly linked to single-target therapy.

In light of these discoveries, as well as our ongoing research on adamantane-based chemotherapeutic agents [34–39], the present study intends to explore a novel class of molecules that combine adamantane with thiazole ring, and look into their potential antibacterial, antifungal, and anti-proliferative properties. Our objective also embraces the crystal structure analysis of typical examples of the new compounds, and the molecular docking investigation, of derivatives that showed the best antibacterial and anti-proliferative activity, using dehydrosqualene synthase from *Staphylococcus aureus* and urokinase-type plasminogen activator from human, as important pharmacological targets.

## Results and discussion

### Chemical synthesis

**Scheme 1:**
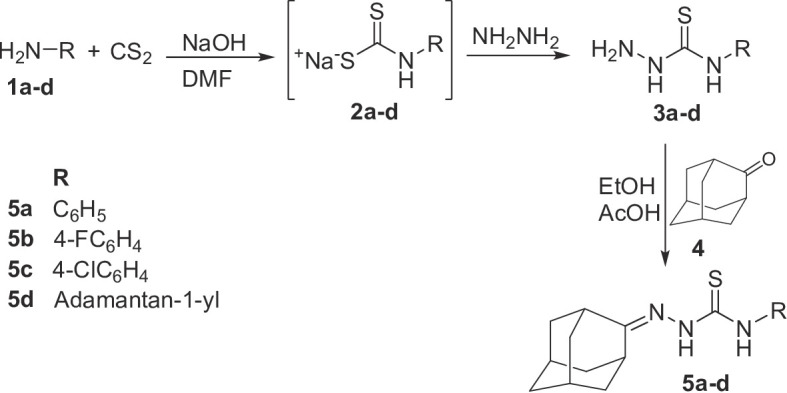
Synthesis of compounds **5a-d**

**Scheme 2:**
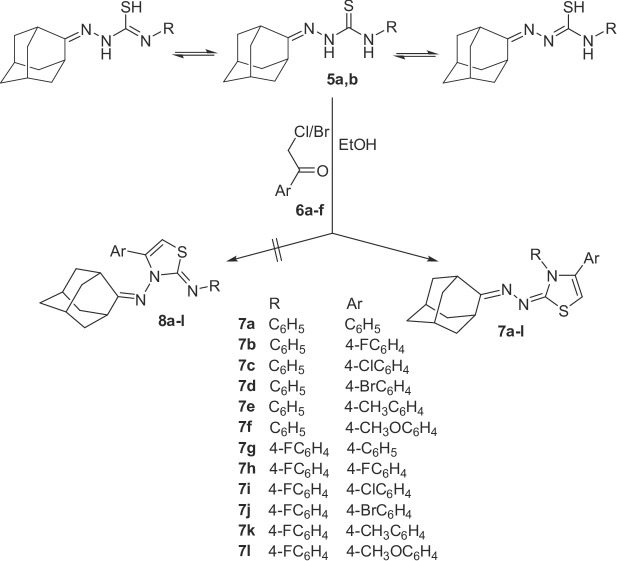
Synthesis of compounds **7a-l**

The designed compounds were synthesized as depicted in Schemes 1 and 2. Thus, the appropriate primary amines **1a-d** were reacted with carbon disulfide, in presence of sodium hydroxide in *N*,*N*-dimethylformamide (DMF) to yield the intermediate sodium aryl or adamantan-1-ylcarbamodithioates **2a-d**, which were reacted with hydrazine to afford the *N*-substituted hydrazinecarbothioamide **3a-d** according to the previously reported procedures [40, 41]. Compounds **3a-d** were subsequently condensed with an equimolar amount of 2-adamantanone **4** in ethanol/acetic acid to afford the target 2-(adamantan-2-ylidene)-*N*-substituted hydrazine-1-carbothioamides **5a-d** in 42–64% yields (Scheme 1, Table 1).

The structures of the 2-(adamantan-2-ylidene)-*N*-substituted hydrazine-1-carbothioamides **5a-d** were assigned based on ^1^H NMR, ^13^C NMR and elemental analysis, which were all consistent with the proposed structures. The ^1^H NMR spectra of compounds **5a-c** showed the presence of two NH protons at *δ* 9.23–9.39 and 8.74–8.79 ppm, whereas the the NH protons of compound **5d** appeared at *δ* 8.14 and 7.47 ppm. The ^13^C NMR spectra of compounds **5a-d** showed the C = S carbons at *δ* 175.58-176.57 ppm, and the C = N carbons at *δ* 161.48-163.49 ppm. In addition, the aromatic and adamantane protons and carbons were clearly observed in their ^1^H NMR and ^13^C NMR spectra (see experimental section).

Compounds **5a** and **5b** were then reacted with various aryl halomethyl ketones [2-bromo-1-phenylethan-1-one, 2-chloro-1-(4-fluorophenyl)ethan-1-one, 2-chloro-1-(4-chlorophenyl)ethan-1-one, 2-bromo-1-(4-bromophenyl)ethan-1-one, 2-bromo-1-(*p*-tolyl)ethan-1-one or 2-bromo-1-(4-methoxyphenyl)ethan-1-one)] **6a-f**, *via* prolonged heating in ethanol, followed by addition of sodium acetate to the cooled reaction mixture, to yield the corresponding (*E*)-2-[(adamantan-2-ylidene)hydrazono]-3,4-diaryl-2,3-dihydrothiazole derivatives **7a-l**, rather than their isomeric counterparts *(Z)-*3-[(adamantan-2-ylidene)amino]-4,*N*-diarylthiazole-2(3*H*)-imines **8a-l**. The structures of the final compounds were assigned based on ^1^H NMR, ^13^C NMR spectra and elemental analysis. In addition, single crystal X-ray analysis of compounds **7a** and **7f** was performed to explore the configuration of compounds **7a-l**, and to exclude the formation of the isomeric compounds **8a-l** (Scheme 2, Table 1).

The main common features of the ^1^H NMR spectra of compounds **7a-l** are the thiazole C-5 protons singlets at *δ* 6.0-6.56 ppm, and the adamantane protons (14 H) as singlets or overlapping multiplets at *δ* 1.53–3.77 ppm. The characteristic ^13^C NMR spectral features of compounds **7a-l** include the presence of the adamantane C = N at *δ* 166.21-172.73 ppm, the thiazole C-2 at *δ* 166-05-167.16, and the thiazole C-5 at 99.41-102.43. In addition, the aromatic and adamantane protons and carbons were clearly observed in their ^1^H NMR and ^13^C NMR spectra (see experimental section).

**Table 1:** Crystallization solvents, melting points, yield percentages, molecular formulae and molecular weights of compounds **5a-d** and **7a-l**


Compound	*R*	Ar	Cryst. Solv.	M.*p*. (^o^C)	Yield (%)	Mol. Formula (Mol. Wt.)
**5a**	C_6_H_5_	-	EtOH	130–132	55	C_17_H_21_N_3_S (299.44)
**5b**	4-FC_6_H_4_	-	EtOH/H_2_O	145–147	64	C_17_H_20_FN_3_S (317.43)
**5c**	4-ClC_6_H_4_	-	EtOH/H_2_O	158–160	55	C_17_H_20_ClN_3_S (333.88)
**5d**	Adamantan-1-yl	-	EtOH/H_2_O	208–210	42	C_21_H_31_N_3_S (357.56)
**7a**	C_6_H_5_	C_6_H_5_	EtOH/H_2_O	200–202	63	C_25_H_25_N_3_S (399.56)
**7b**	C_6_H_5_	4-FC_6_H_4_	EtOH/H_2_O	180–182	67	C_25_H_24_FN_3_S (417.55)
**7c**	C_6_H_5_	4-ClC_6_H_4_	EtOH/H_2_O	214–216	69	C_25_H_24_ClN_3_S (434.00)
**7d**	C_6_H_5_	4-BrC_6_H_4_	EtOH/H_2_O	188–190	93	C_25_H_24_BrN_3_S (478.45)
**7e**	C_6_H_5_	4-CH_3_C_6_H_4_	EtOH/H_2_O	196–198	59	C_26_H_27_N_3_S (413.58)
**7f**	C_6_H_5_	4-CH_3_OC_6_H_4_	EtOH/H_2_O	188–190	65	C_26_H_27_N_3_OS (429.58)
**7 g**	4-FC_6_H_4_	C_6_H_5_	EtOH	195–197	96	C_25_H_24_FN_3_S (417.55)
**7 h**	4-FC_6_H_4_	4-FC_6_H_4_	EtOH	196–198	60	C_25_H_23_F_2_N_3_S (435.54)
**7i**	4-FC_6_H_4_	4-ClC_6_H_4_	EtOH	150–152	89	C_25_H_23_ClFN_3_S (451.99)
**7j**	4-FC_6_H_4_	4-BrC_6_H_4_	EtOH	198–200	94	C_25_H_23_BrFN_3_S (496.44)
**7k**	4-FC_6_H_4_	4-CH_3_C_6_H_4_	EtOH	138–140	88	C_26_H_26_FN_3_S (431.57)
**7 L**	4-FC_6_H_4_	4-CH_3_OC_6_H_4_	EtOH	178–180	90	C_26_H_26_FN_3_OS (447.57)

### Single crystal XRD study and structural insights

#### Structural description of compounds 5c, 7a, and 7f and intermolecular contacts

**Fig. 3:**
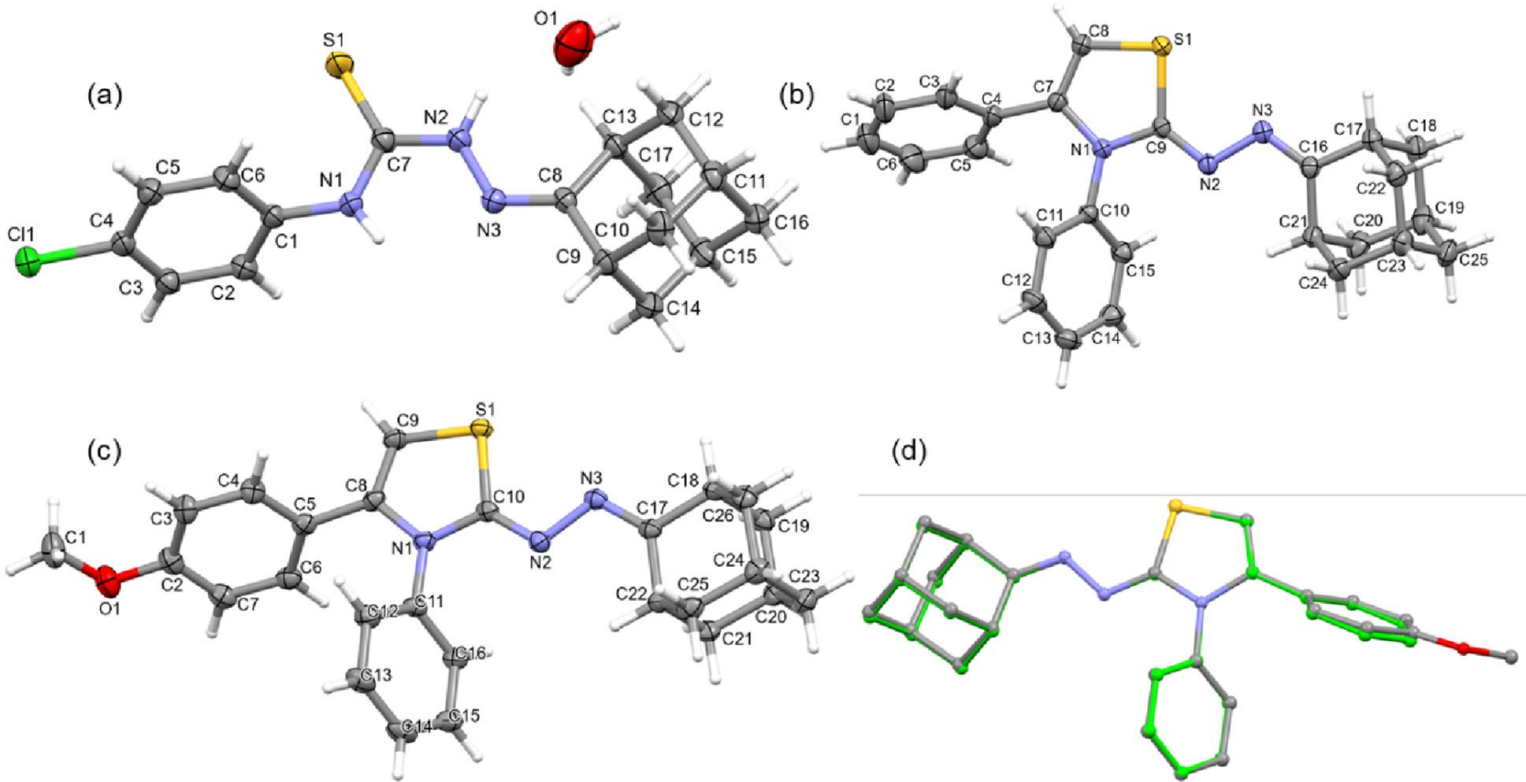
ORTEP representation of (**a**) compound **5c** with a lattice water molecule, (**b**) compound **7a**, (**c**) compound **7f**, all shown with 40% probability ellipsoids, and (d) structural overlay of compounds **7a** (green) and **7f** (grey)

Single-crystal X-ray diffraction analysis was performed to determine the crystal structures of compounds **5c**, **7a** and **7f**. X-ray analysis revealed that compound **5c** crystallizes in the monoclinic system with space group *C*2/c. In the crystal lattice, a single water molecule was located in a stoichiometric ratio of 1:0.06. Compounds **7a** and **7f** also crystallize in the monoclinic system, with space group *P*2_1_/c and *P*2_1_, respectively. The asymmetric units of the latter compounds each contain a single molecule. Crystal data and refinement parameters for these compounds are summarized in Table 2. The ORTEP representations are illustrated in Fig. 3a-c. In all three structures, the six-membered rings of the adamantyl moiety exhibit the typical chair conformation. Compounds **7a** and **7f** differ by the presence of a methoxy substituent on the phenyl ring. Structural superimposition of these two compounds shows an overall good fit (Fig. 3d). In compound **5c**, the thiourea moiety adopts two different conformations, namely *anti* and *syn*, with respect to the amine H and S atoms (due to rotation about the C–N bond). This feature has also been previously reported in closely related structures [42].

**Table 2:** Crystal data and structure refinement parameters of compounds **5c**, **7a** and **7f**


	Compound 5c	Compound 7a	Compound 7f
CCDC number	2,477,371	2,477,374	2,477,375
Empirical formula	C_17_H_20_ClN_3_S.H_2_O	C_25_H_25_N_3_S	C_26_H_27_N_3_OS
Formula weight	334.92	399.54	429.56
Temperature (K)	160	160	160
Crystal system	Monoclinic	Monoclinic	Monoclinic
Space group	*C*2/c	*P*2_1_/c	*P*2_1_
*a*/Å	24.1495 (3)	10.82409 (12)	11.0164 (1)
*b*/Å	6.4705 (1)	6.51732 (9)	6.5217 (1)
*c*/Å	21.2674 (3)	30.0707 (3)	15.6370 (2)
*α*/°	90	90	90
*β*/°	102.890 (1)	96.7639 (9)	101.434 (1)
*γ*/°	90	90	90
Volume/Å^3^	3239.49 (8)	2106.55 (4)	1101.15 (2)
*Z*	8	4	2
Calculated density (g/cm^3^)	1.373	1.260	1.296
Absorption coefficient (mm^− 1^)	3.282	1.472	1.480
*F*(000)	1413.0	848.0	456.0
Crystal size (mm^3^)	0.23 × 0.17 × 0.13	0.21 × 0.1 × 0.03	0.23 × 0.11 × 0.03
Radiation	Cu Kα (*λ* = 1.54184)	Cu Kα (*λ* = 1.54184)	Cu Kα (*λ* = 1.54184)
2Θ range for data collection	7.51 to 152.42	5.92 to 148.952	5.766 to 149.006
Index ranges	-30 ≤ *h* ≤ 29, -8 ≤ *k* ≤ 7, -26 ≤ *l* ≤ 26	-13 ≤ *h* ≤ 13, -8 ≤ *k* ≤ 8, -37 ≤ *l* ≤ 34	-11 ≤ *h* ≤ 13, -8 ≤ *k* ≤ 8, -19 ≤ *l* ≤ 19
Reflections collected	17,810	22,852	22,613
Independent reflections	3374 [*R*_int_ = 0.0359, *R*_sigma_ = 0.0192]	4288 [*R*_int_ = 0.0227, *R*_sigma_ = 0.0190]	4485 [*R*_int_ = 0.0392, *R*_sigma_ = 0.0292]
Data/restraints/parameter	3374/2/213	4288/0/263	4485/1/281
Goodness-of-fit on *F*^2^	1.065	1.061	1.046
Final *R* indices [*I > 2* σ*(I)*]	*R*_1_ = 0.0354, *wR*_2_ = 0.0919	*R*_1_ = 0.0433, *wR*_2_ = 0.1078	*R*_1_ = 0.0342, *wR*_2_ = 0.0857
Final *R* indices (all data)	*R*_1_ = 0.0382, *wR*_2_ = 0.0942	*R*_1_ = 0.0483, *wR*_2_ = 0.1155	*R*_1_ = 0.0373, *wR*_2_ = 0.0881
Largest diff. peak and hole (e.Å^−3^)	0.37/-0.28	0.49/-0.29	0.14/-0.34

In compound **5c**, the C8 = N3 distance is found to be 1.281(2) Å, which is comparable to that observed in a similar structure, (*R*)-camphor-4-phenylthiosemicarbazone (1.283 Å) [43]. The corresponding distances in compounds **7a** (1.285(2) Å) and **7f** (1.284(3) Å) are also very similar, confirming the presence of a double bond. Similarly, the C9 = N2 distance in compound **7a** was found to be 1.286(2) Å, and the corresponding bond C10 = N2 distance in compound **7f** is 1.288(3) Å, also indicating a comparable double bond length. The central fragment comprising atoms C1, N1, C7, N2, N3 and C8 in **5c** makes a dihedral angle of 69.15° with respect to the plane of the substituted phenyl ring. In compound **7a**, the phenyl ring attached to the N atom of the central thiazole ring is oriented at an angle of 54.99°, while the corresponding angle in compound **7f** is 53.48°. Similarly, the dihedral angle between the mean planes of the thiazole and the phenyl ring attached to the C atom of the thiazole ring is 59.39° in compound **7a** and 60.41° in compound **7f**. The attached phenyl rings are oriented at angles of 61.73° in compound **7a** and 65.21° in compound **7f**. These observations clearly indicate that the molecular conformation of compounds **7a** and **7f** are very similar, irrespective of the methoxy substituent introduced in compound **7f**.

**Fig. 4:**
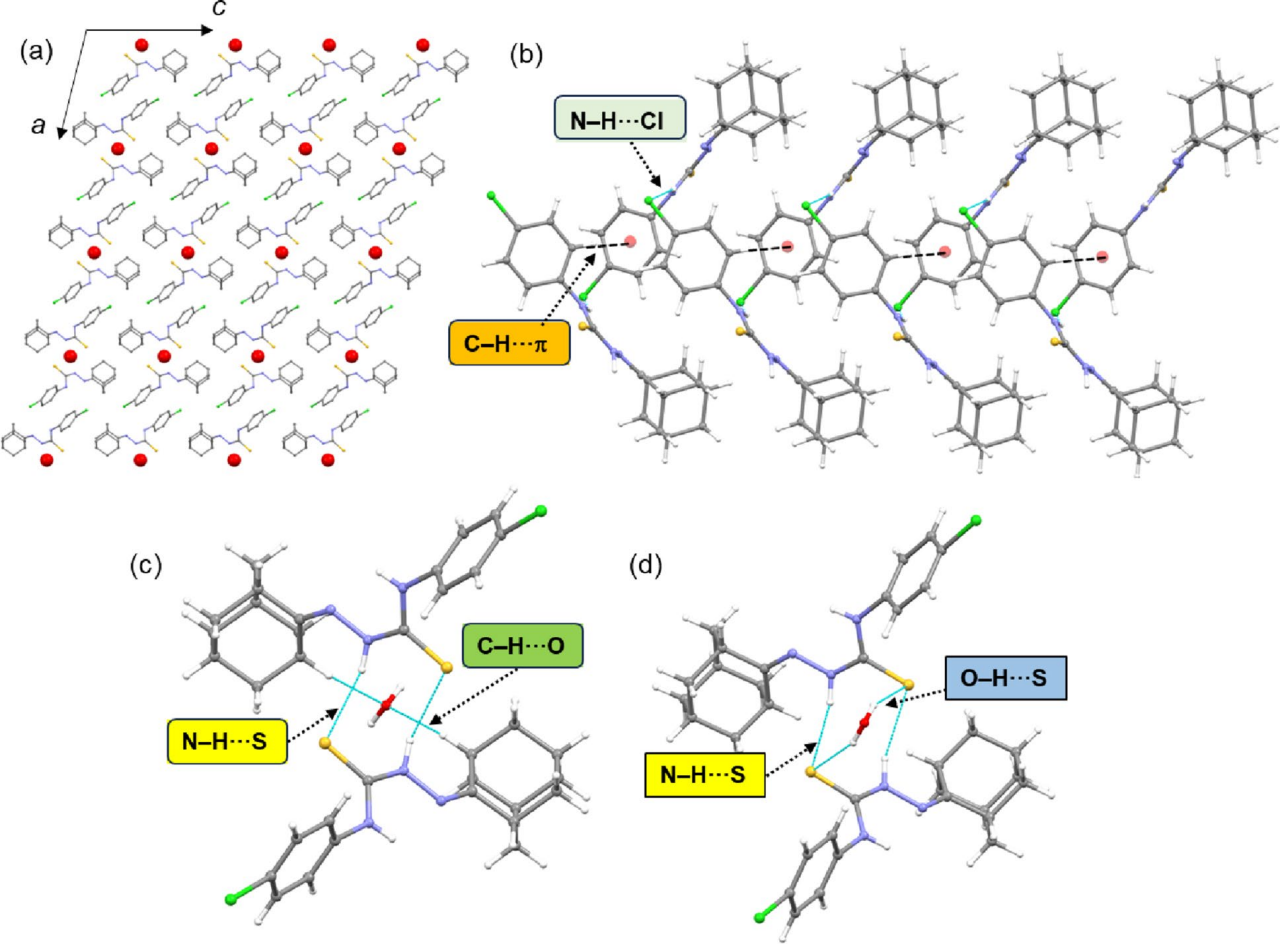
(**a**) Layered architecture of compound **5c** projected onto the crystallographic *ac* plane; spheres represent lattice water molecules located between molecular layers. H atoms are omitted for clarity, (**b**) supramolecular chain of **5c** generated by N–H···Cl and C–H···π interactions, and two different dimeric motifs formed by (**c**) N–H···S/C–H···O interactions, and (**d**) N–H···S/O–H···S interactions

The intermolecular interactions that stabilize the crystal structure of compound **5c** are summarized in Table 3. Molecules of compound **5c** form layered structures, with lattice water molecules located between the layers in the solid state (Fig. 4a). A wide range of intermolecular interactions is present in the crystal structure of compound **5c**, including N–H···Cl, N–H···S, O–H···S, C–H···Cl, C–H···O and C–H···π interactions. Adjacent molecules are linked by N–H···Cl hydrogen bonds and C–H···π interactions, forming molecular chains that run parallel to the crystallographic *b-*axis (Fig. 4b). Molecules in one layer of **5c** are connected to those in another layer via a lattice water molecule through C–H···O interactions (Fig. 4c). These pairs of molecules are also interconnected by N–H···S hydrogen bonds, forming an $\:{R}_{2}^{2}\left(8\right)$ motif, as observed in closely related structures [42, 44]. Additionally, N–H···S and O–H···S hydrogen bonds generate another dimeric motif, as shown in Fig. 4d. Intermolecular C–H···Cl interactions also contribute to linking adjacent molecules of compound **5c** into chains running parallel to the *b* axis.

**Table 3:** Intermolecular interactions (Å, °) in compound **5c**

	H···A	D···A	∠D–H···A
N1–H1···Cl1^i^	2.85 (2)	3.588 (2)	149.2 (19)
C2–H2A···*Cg*1^i^	2.90	3.622 (2)	134
C13–H13···O1	2.24	3.090 (5)	142
N2–H2···S1^ii^	2.65 (3)	3.404 (2)	143 (2)
C14–H14B···Cl1^ii^	2.85	3.626 (3)	135

Symmetry elements: (i) 3/2-*x*, ½+*y*, ½-*z*; (ii) 1-*x*, *y*, ½-*z* ; (iii) 3/2-*x*, 3/2 + *y*, ½-*z*. *Cg*1: centroid of the ring containing atoms C1-C6

**Fig. 5:**
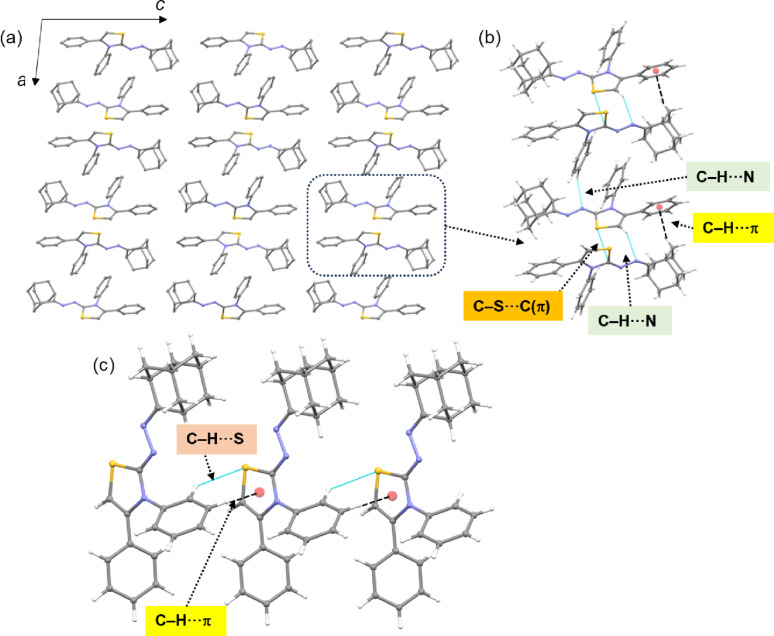
(**a**) Layered architecture and columnar arrangement of compound **7a** projected onto the crystallographic *ac* plane; the basic structural motif is highlighted with a dashed box, (**b**) adjacent structural motifs are connected by C–H···N interactions and (**c**) supramolecular chain is formed through C–H···π and C–H···S interactions

The intermolecular interactions that stabilize the crystal structure of compound **7a** are summarized in Table 4. Molecules of compound **7a** form layered structures and can also be described as being arranged in a columnar fashion along the crystallographic *ac* plane (Fig. 5a). The intermolecular C–H···N, C–H···S, C–H···π interactions, along with chalcogen bonding (S1···C9) contribute to the stabilization of the crystal structure. Figure 5b shows the molecular dimer of compound **7a** (basic structural motif), which is stabilized by intermolecular C–H···N interactions, supported by C–H···π and a σ-hole interaction (chalcogen bonding) involving the sulfur atom (S1) and one of the carbon atoms of the thiazole ring. The geometrical parameters suggest that this represents a plausible chalcogen bonding interaction. Furthermore, adjacent structural motifs are interconnected through C–H···N interactions involving atoms H13 and N2. Molecules arranged along the *a* axis are interlinked in this manner in the solid state. The combined C–H···S and C–H···π interactions link the adjacent molecules into a supramolecular chain, as shown in Fig. 5c.

**Table 4:** Intermolecular interactions (Å, °) in compound **7a**

	H···A	D···A	∠D–H···A
C8–H8···N3^i^	2.60	3.437 (2)	147
C9–S1···C9^i^	3.403 (2)		159.88 (6)
C18–H18A···*Cg*1^ii^	2.76	3.634 (2)	147
C13–H13···N2^iii^	2.51	3.271 (2)	137
C15–H15···S1^iv^	2.91	3.686 (2)	140
C14–H14···*Cg*2^iv^	2.93	3.561 (2)	125

Symmetry elements: (i) -*x*, -½+*y*, ½-*z*; (ii) -*x*, ½+*y*, ½-*z*; (iii) 1-*x*, ½+*y*, ½-*z*; (iv) *x*, 1 + *y*, *z*; *Cg*1: centroid of the phenyl ring (C1-C6) and *Cg*2: centroid of the thiazole ring

**Fig. 6:**
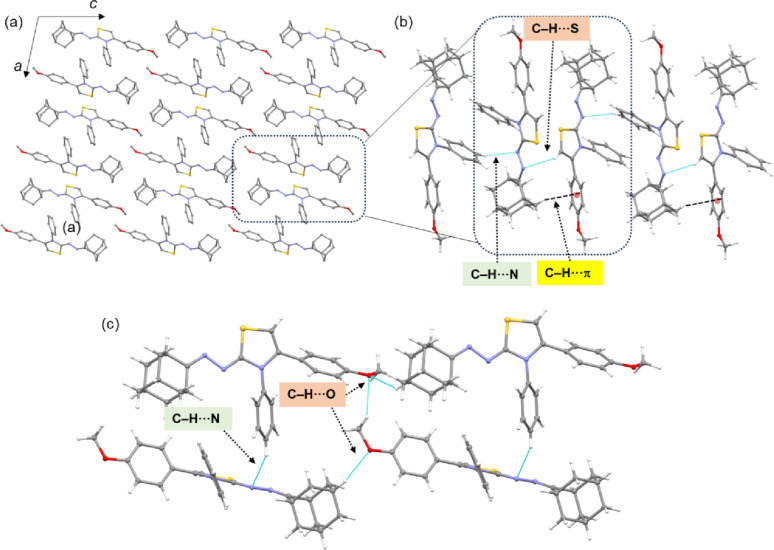
(**a**) Layered architecture and columnar arrangement of compound **7f** projected onto the crystallographic *ac* plane; the basic structural motif is highlighted with a dashed box, (**b**) adjacent structural motifs are interconnected by C–H···N interactions and (**c**) C–H···O interactions connect molecules within the same layer as well as between adjacent layers, while C–H···N interactions also interconnect adjacent layers

The intermolecular interactions that stabilize the crystal structure of compound **7f** are summarized in Table 5. The crystal packing of compound **7f** is very similar to that of compound **7a**, as shown in Fig. 6a. However, both invariant and variant interactions are observed between the two structures. For instance, the basic structural motif formed in compounds **7a** and **7f** is very similar, but its stabilization differs slightly between the two compounds. In compound **7f**, this motif is stabilized by C–H···N (involving atoms H9 and N3) and C–H···π (involving atoms H19A and the centroid of the methoxy phenyl ring) interactions (Fig. 6b). The chalcogen bond present in compound **7a** is absent in this motif. However, the adjacent structural motifs in compound **7f** are interconnected by C–H···N interactions, similar to those in compound **7a**. The methoxy group in compound **7f** participates in C–H···O interactions as both a donor and an acceptor. One such interaction, involving the adamantyl group and an oxygen atom, links adjacent molecules within the same layer into a chain. Another C–H···O interaction (between the methoxy-methyl group and methoxy oxygen) interlinks molecules in one layer with those in the adjacent layer. This interlayer connection is further supported by an additional C–H···N interaction (involving atom H14 and N2), as shown in Fig. 6c. Another common structural feature observed in both compounds **7a** and **7f** is the formation of a supramolecular chain through intermolecular C–H···S and C–H···π interactions, similar to that depicted in Fig. 5c.

**Table 5:** Intermolecular interactions (Å, °) in compound **7f**

	H···A	D···A	∠D–H···A
C1–H1···O1^i^	2.50	3.243 (4)	132
C24–H24···O1^ii^	2.53	3.305 (3)	135
C14–H14···N2^iii^	2.55	3.330 (3)	139
C9–H9···N3^iv^	2.68	3.515 (3)	147
C19–H19A···*Cg*1^v^	2.59	3.471 (2)	148
C16–H16···S1^vi^	2.98	3.763 (2)	141
C15–H15···*Cg*2^vi^	2.99	3.613 (3)	125

Symmetry: (i) 1-*x*, ½+*y*, 2-*z*; (ii) *x*, *y*, -1 + *z*; (iii) 1-*x*, -½+*y*, 2-*z*; (iv) -*x*, ½+*y*, 1-*z*; (v) -*x*, -½+*y*, 1-*z*; (vi) *x*, -1 + *y*, *z*. *Cg*1: centroid of the methoxy phenyl ring (C2-C7) and *Cg*2: centroid of the thiazole ring

### In vitro antibacterial and antifungal activities

The in vitro antibacterial and antifungal activities of compounds **5a-d**, and **7a-l** were assessed against a panel of standard pathogenic bacterial and fungal strains obtained from the Institute of Fermentation of Osaka (IFO) namely; *Staphylococcus aureus* IFO 3060, *Bacillus subtilis* IFO 3007 and *Micrococcus luteus* IFO 3232 (Gram-positive bacteria), *Escherichia coli* IFO 3301 and *Pseudomonas aeruginosa* IFO 3448 (Gram-negative bacteria), and the pathogenic fungi *Candida albicans* IFO 0583, *Aspergillus oryzae* IFO 4177 and *Aspergillus niger* IFO 4414. The preliminary evaluation was carried out using the semi-quantitative agar disc-diffusion method using Müller-Hinton agar medium [45]. The outcomes of the preliminary antimicrobial screening of compounds **5a-d**, and **7a-l** (200 µg/disc) are summarized in Table 6, along with the reference drugs ampicillin trihydrate, ciprofloxacin, and fluconazole (100 µg/disc).

**Table 6:** In vitro antibacterial and antifungal activities of compounds **5a-d** and **7a-l** (200 µg/8 mm disc), the broad-spectrum antibacterial drugs ampicillin trihydrate, Ciprofloxacin and the antifungal drug fluconazole (100 µg/8 mm disc) against *Staphylococcus aureus* IFO 3060 (SA), *Bacillus subtilis* IFO 3007 (BS) and *Micrococcus luteus* IFO 3232 (ML), the Gram-negative bacterial strains *Escherichia coli* IFO 3301 (EC) and *Pseudomonas aeruginosa* IFO 3448 (PA) and the standard fungi *Candida albicans* IFO 0583 (CA), *Aspergillus oryzae* IFO 4177 (AO) and *Aspergillus Niger* IFO 4414 (AN)


Compound	*R*	Ar	Diameter of Inhibition Zone (mm)^1^							
			SA	BS	ML	EC	PA	CA	AO	AN
**5a**	C_6_H_5_	-	**25 (4.2)**	**28 (2.8)**	-	15	-	-	-	-
**5b**	4-FC_6_H_4_	-	**27 (3.2)**	**31 (1.8)**	15	15	-	-	-	-
**5c**	4-ClC_6_H_4_	-	**28 (3.0)**	**30 (2.4)**	14	16	-	-	-	-
**5d**	Adamantan-1-yl	-	-	11	-	-	-	-	-	-
**7a**	C_6_H_5_	C_6_H_5_	**22 (6.5)**	**25 (4.8)**	-	15	-	-	-	-
**7b**	C_6_H_5_	4-FC_6_H_4_	14	15	-	-	-	-	-	-
**7c**	C_6_H_5_	4-ClC_6_H_4_	14	15	-	-	-	-	-	-
**7d**	C_6_H_5_	4-BrC_6_H_4_	18	14	-	-	-	-	-	-
**7e**	C_6_H_5_	4-CH_3_C_6_H_4_	15	17	10	12	-	-	-	-
**7f**	C_6_H_5_	4-CH_3_OC_6_H_4_	**26**	**28**	-	18	-	-	-	-
**7g**	4-FC_6_H_4_	C_6_H_5_	15	18	-	-	-	-	-	-
**7h**	4-FC_6_H_4_	4-FC_6_H_4_	10	-	-	-	-	-	-	-
**7i**	4-FC_6_H_4_	4-ClC_6_H_4_	**28 (3.6)**	**34 (2.1)**	15	**22 (4.5)**	-	-	-	-
**7j**	4-FC_6_H_4_	4-BrC_6_H_4_	12	-	-	-	-	-	-	-
**7k**	4-FC_6_H_4_	4-CH_3_C_6_H_4_	**20**	**21**	-	15	-	-	-	-
**7 L**	4-FC_6_H_4_	4-CH_3_OC_6_H_4_	18	16	-	-	-	-	-	-
Ampicillin trihydrate			**28 (2.0)**	**30 (1.0)**	**25 (1.5)**	**24 (2.0)**	**22 (8.0)**	NT	NT	NT
Ciprofloxacin			**34 (0.75)**	**38 (0.5)**	**32 (1.0)**	**38 (0.5)**	**36 (1.0)**	NT	NT	NT
Fluconazole			NT	NT	NT	NT	NT	**21**	**22**	**24**

^1^ (-): Inactive (inhibition zone < 10 mm), figures shown in parentheses represent the MIC values (µg/mL), NT: not tested

In general, the results indicate varying levels of inhibition for the synthesized compounds against the tested organisms. Compounds **5a**, **5b**, **5c**, **7a**, **7f**, **7i** and **7k** displayed marked activity against the Gram-positive bacteria *S. aureus* and *B. subtilis* (growth inhibition zones > 20 mm). Meanwhile, compounds **5b** and **7i** retained moderate activity (growth inhibition zones 15–20 mm) against *M. luteus*. Regarding the activity against the tested Gram-negative bacteria, compound **7i** showed potent activity against *E. coli*, while compounds **5a**, **5b**, **5c**, **7a**, **7f** and **7k** displayed moderate activity. In addition, all the compounds were completely inactive against the Gram-negative bacteria *P. aeruginosa* and all the tested fungal strains (growth inhibition zones < 10 mm).

The minimum inhibitory concentrations (MIC) of the highly active compounds **5a**, **5b**, **5c**, **7a**, **7f**, **7i** and **7k** were determined using the microdilution susceptibility method in Müller-Hinton Broth [46] and were found to be less active than the reference drugs, and thus consistent with the findings of the primary screening (Table 6).

At the moment, it is challenging to infer a distinct structure-activity relationships (SAR). However, the three adamantanylidene-*N*-aryl hydrazinecarbothioamides (**5a**, **5b**, **5c)** clearly showed superior efficacy in comparison to their *N*-adamantyl equivalents. As regards the adamantane-thiazole hybrids, the nature of substitutions at N3 and C4 of the thiazole ring does not allow a clear-cut correlation with the antibacterial activity. Four compounds with various substitution patterns (**7a**, **7f**, **7i**, **7k**) showed activity; and the molecule with two halophenyl moieties (**7i**) was the most effective.

### In vitro anti-proliferative activity

The in vitro anti‑proliferative action of compounds **5a-d** and **7a-l** was evaluated using the MTT colorimetric assay [47, 48] against five human tumor cell lines: Human prostate cancer (PC3), Epithelioid Carcinoma Cervix cancer (HeLa), Hepatocellular carcinoma (HepG-2), Colorectal carcinoma Colon cancer (HCT-116), and Mammary gland breast cancer (MCF-7). The cell lines were obtained from the Holding company for biological products and vaccines (VACSERA), Cairo, Egypt.

The effect was expressed as IC_50_ values (µM), which indicate concentration required to inhibit cell growth by 50%. In this study, IC_50_ values were categorized as follows: 1–10 µM (very strong), 11–20 µM (strong), 21–50 µM (moderate), 51–100 µM (weak), and > 100 µM (non-cytotoxic). Doxorubicin [49] and Sorafenib [50] were used as reference drugs. Table 7 presents results of the anti-proliferative activity of compounds **5a-d** and **7a-l**, as well as the anticancer drugs Doxorubicin and Sorafenib.

The standard drugs Doxorubicin and Sorafenib displayed IC_50_ values of 4.17–8.87 µM and 5.47–11.53 µM, respectively. Adamantane derivatives with hydrazine-carbothioamide chain (**5a**, **5b**, **5c**) were the highest active among the tested compounds. They presented significant anti-proliferative activity, with IC_50_ values ranging from 4.90 µM to 16.40 µM, categorizing them as comparable in activity to that of reference drugs. Regarding the adamantane-thiazole hybrids, derivatives **7f** and **7 L** exhibited superior activity compared to their equivalents, especially against the tested liver and breast (HepG-2 and MCF-7) cancer cell lines (IC_50_ < 25 µM). The superior performance of these two molecules compared to other thiazole compounds might be attributed to the presence of a *p*-methoxyphenyl substituent at position 4 of the heterocyclic ring. It is worth mentioning that the presence of a 4-methoxyphenyl substituent in one of our previously reported classes of adamantane-linked compounds [36] was found optimal for the anti-proliferative activity. Taken together, the perspective of having a thiazole ring in the molecule can be further explored by future design of new methoxyphenyl- and similarly substituted analogues.

**Table 7:** In vitro anti-proliferative activity of the tested compounds **4a-d**, **7a-l**, the reference anticancer drugs doxorubicin and Sorafenib, expressed as IC_50_ values against PC-3, HeLa, HepG-2, HCT-116, and MCF-7 cancer cell lines


Compound	*R*	Ar	IC_50_ (µM)^1^				
			PC-3	HeLa	HepG-2	HCT-116	MCF-7
**5a**	C_6_H_5_	-	**13.62 ± 1.0**	**8.43 ± 0.6**	**4.90 ± 0.2**	**6.78 ± 0.4**	**5.81 ± 0.3**
**5b**	4-FC_6_H_4_	-	**14.48 ± 1.1**	**10.80 ± 0.9**	**7.93 ± 0.4**	**9.46 ± 0.8**	**8.60 ± 0.5**
**5c**	4-ClC_6_H_4_	-	**16.40 ± 1.3**	**12.62 ± 1.1**	**9.80 ± 0.9**	**11.6 ± 1.2**	**10.22 ± 0.9**
**5d**	Adamantan-1-yl	-	> 100	> 100	88.02 ± 4.1	˃100	90.19 ± 4.9
**7a**	C_6_H_5_	C_6_H_5_	> 100	> 100	91.55 ± 4.6	˃100	83.98 ± 4.0
**7b**	C_6_H_5_	4-FC_6_H_4_	85.51 ± 4.2	89.30 ± 4.6	60.42 ± 3.5	79.65 ± 3.9	˃100
**7c**	C_6_H_5_	4-ClC_6_H_4_	58.76 ± 3.2	54.87 ± 3.2	44.57 ± 2.5	46.37 ± 2.8	49.17 ± 2.8
**7d**	C_6_H_5_	4-BrC_6_H_4_	67.10 ± 3.5	63.37 ± 3.5	49.56 ± 2.9	57.61 ± 3.2	52.06 ± 3.0
**7e**	C_6_H_5_	4-CH_3_C_6_H_4_	72.85 ± 3.8	77.83 ± 3.8	53.47 ± 3.1	67.62 ± 3.6	47.30 ± 2.7
**7f**	C_6_H_5_	4-CH_3_OC_6_H_4_	29.89 ± 1.9	41.97 ± 2.4	**19.76 ± 1.5**	33.41 ± 2.1	**17.17 ± 1.3**
**7 g**	4-FC_6_H_4_	C_6_H_5_	75.17 ± 3.8	68.43 ± 3.6	55.15 ± 3.3	62.54 ± 3.4	59.36 ± 3.4
**7 h**	4-FC_6_H_4_	4-FC_6_H_4_	˃100	93.44 ± 5.1	72.30 ± 3.8	82.75 ± 4.1	78.21 ± 3.7
**7i**	4-FC_6_H_4_	4-ClC_6_H_4_	51.88 ± 2.9	74.01 ± 3.8	39.02 ± 2.3	64.95 ± 3.5	42.73 ± 2.5
**7j**	4-FC_6_H_4_	4-BrC_6_H_4_	76.20 ± 3.9	80.18 ± 4.0	58.21 ± 3.4	71.16 ± 3.7	65.78 ± 3.5
**7k**	4-FC_6_H_4_	4-CH_3_C_6_H_4_	79.14 ± 4.0	84.22 ± 4.2	59.01 ± 3.4	74.51 ± 3.7	63.80 ± 3.5
**7 L**	4-FC_6_H_4_	4-CH_3_OC_6_H_4_	45.54 ± 2.6	38.79 ± 2.2	34.66 ± 2.1	30.17 ± 1.9	**24.51 ± 1.7**
Doxorubicin			**8.87 ± 0.6**	**5.57 ± 0.4**	**4.50 ± 0.2**	**5.23 ± 0.3**	**4.17 ± 0.2**
Sorafenib			**11.53 ± 0.9**	**8.04 ± 0.5**	**9.18 ± 0.6**	**5.47 ± 0.3**	**7.26 ± 0.3**

^1^ IC_50_ values presented as the mean ± SD of three separate determinations

### Molecular Docking analysis

It has been observed that inhibition of dehydrosqualene synthase (*Sa*CrtM) from *Staphylococcus aureus* not only prevents the production of the virulence factor staphyloxanthin, but also disrupts biofilm formation in *S. aureus*, thereby enhancing bacterial clearance by the host immune system [51]. Using the crystal structure of *Sa*CrtM complexed with a novel di-alkylated ethylenediamine bearing an adamantyl head group and a geranyl side chain (ligand ID: RWZ) [52], we performed molecular docking analysis with the most potent antibacterial compounds, **7f** and **7i**. The docking score were compared with that of the co-crystallized inhibitor, RWZ. Prior to the docking simulation, the co-crystallized inhibitor was re-docked with *Sa*CrtM to validate the predictive ability of the AutoDock Vina program. The results showed that the predicted pose of the co-crystallized inhibitor closely matched its crystallographic conformation, with a docking score of -6.7 kcal/mol. Compounds **7f** and **7i** showed docking scores of -10.1 and − 9.4 kcal mol, respectively, indicating stronger predicted binding affinities than RWZ.

**Fig. 7:**
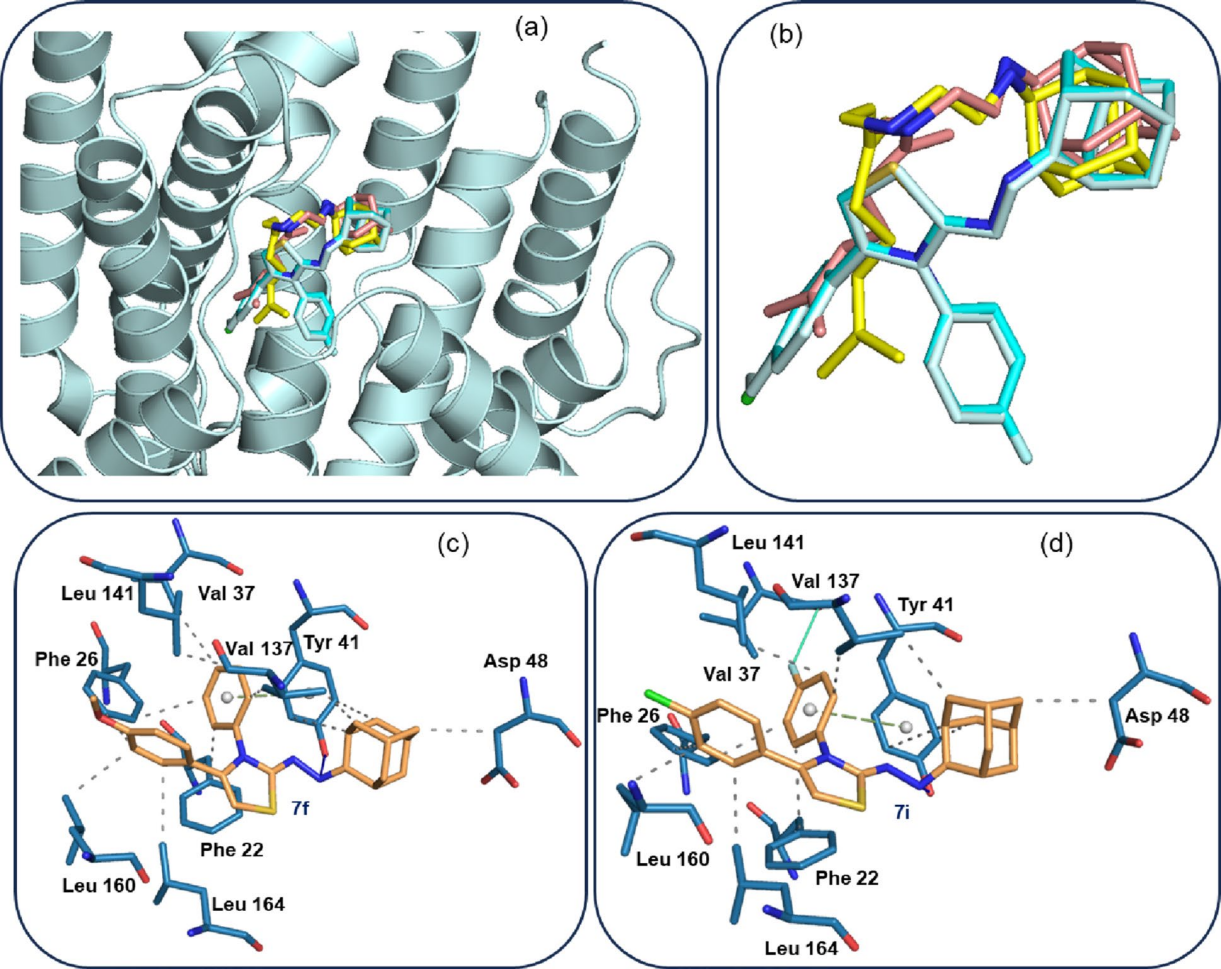
(**a**) Cartoon representation of *Sa*CrtM with the co-crystallized inhibitor (X-ray: yellow; Predicted: brown) and predicted binding poses of compounds **7f** (white) and **7i** (cyan), (**b**) close-up view of the small molecules’ poses at the active site, (**c**) interactions between active site resides and compound **7f** and (**d**) interactions between active site residues and compound **7i**

Figure 7 shows the binding poses of compounds **7f**, **7i**, as well as the X-ray and predicted conformations of the co-crystallized inhibitor RWZ. As shown, the adamantyl groups of compounds **7f** and **7i** occupy the same position as that of RWZ. Additionally, the predicted poses of **7f** and **7i** are highly similar to each other. Protein-ligand interaction analysis revealed that key residues of *Sa*CrtM participate in interactions with both compounds **7f** and **7i** (Fig. 7c, d). Because the predicted poses of **7f** and **7i** are very similar, the interacting residues are largely the same, with only minor differences observed. The side chain of Tyr 41 forms a hydrogen bond with the nitrogen atom attached to the adamantyl moiety *via* an O–H···N interaction in both complexes. In the **7f** complex, residues Tyr 41, Asp 48, and Val 137 form hydrophobic interactions with the adamantyl group. The unsubstituted phenyl ring of **7f** engages in π-π stacking with Tyr 41 and hydrophobic interactions with Phe 22, Phe 26, Lav 37, Val 137, and Leu 141. The methoxyphenyl ring also forms hydrophobic interactions with Phe 26, Leu 160, Leu 164 residues. The central thiazole ring does not participate in any interactions with active site residues. In the **7i** complex, the intermolecular interactions are very similar to those observed in the **7f** complex. One additional interaction (F···O) is observed between the backbone oxygen atom of Val 37 and the F atom of compound **7i**. This analysis clearly suggests that compounds **7f** and **7i** have the potential to inhibit the *Sa*CrtM protein, in agreement with the in vitro results.

**Fig. 8:**
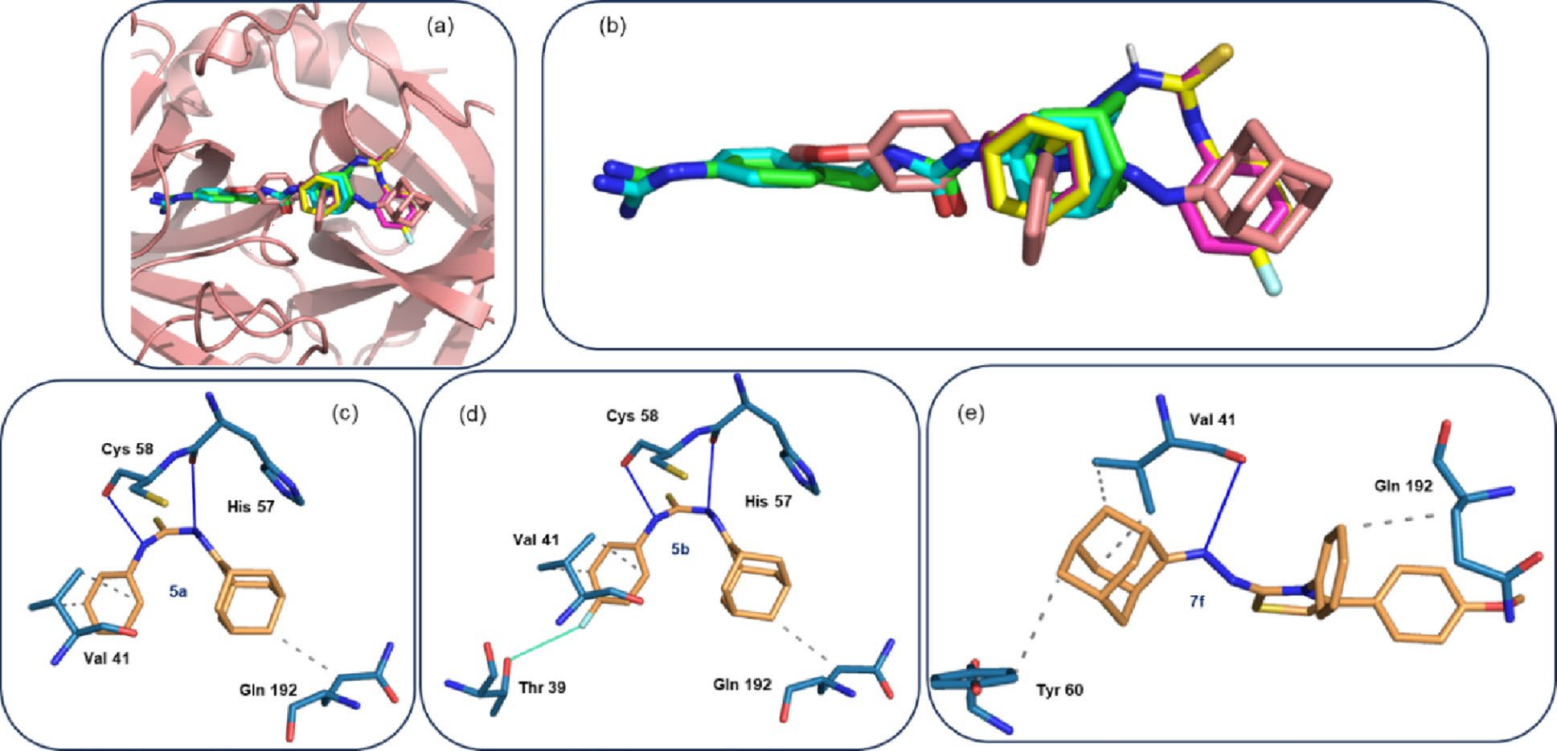
(**a**) Cartoon representation of the human urokinase-type plasminogen activator showing the co-crystallized inhibitor (X-ray: cyan; predicted: green) and the predicted binding poses of compounds **5a** (magenta), **5b** (yellow) and **7f** (brown), (**b**) close-up view of the small molecule poses at the active site, (**c**) interactions between active site resides and compound **5a**, (**d**) interactions between active site residues and compound **5b**, and (**e**) interactions between active site residues and compound **7f**

Similarly, the most potent anti-proliferative compounds (**5a**, **5b**, and **7f**) were subjected to molecular docking analysis against a cancer-related target, urokinase-type plasminogen activator receptor (uPAR) [53, 54]. The results indicated that these compounds occupy the position of the co-crystallized adamantyl derivative (AGB) [55]. The docking score for the co-crystallized inhibitor was − 6.7 kcal/mol. Compounds **5a** and **5b** exhibited similar binding modes at the active site, with overlap between the adamantyl moiety of the co-crystallized inhibitor and that of **5a** and **5b** (Fig. 8a, b). The docking scores for compounds **5a** and **5b** were − 6.7 and − 7.1 kcal/mol, respectively, which are comparable to that of AGB. Interestingly, compound **7f** showed a different binding pose at the active site, in which the central thiazole ring occupies the position of the adamantyl moiety of AGB, with a docking score of -7.2 kcal/mol.

To gain further insights into the interactions between the active site residues of the target and the potent compounds, we performed protein-ligand interaction analysis based on the predicted poses (Figs. 8c-e). In the **5a** complex, the backbone oxygen atoms of His 57 and Cys 58 form hydrogen bonds (N–H···O = C) with the amine NH groups of the thiourea moiety. Similar interactions are also present in the **5b** complex, as both compounds bind in a similar manner at the active site. Residue Val 41 forms hydrophobic interactions with the phenyl ring, while Gln 192 interacts hydrophobically with the adamantyl moiety. Notably, an additional O–H···F interaction is observed between the OH group of Thr 39 and the fluorine atom of **5b**. In the **7f** complex, Val 41 and Tyr 60 form hydrophobic interactions with the adamantyl moiety. The backbone oxygen atom of Val 41interacts with the nitrogen atom attached to the adamantyl moiety. Residue Gln 192 also forms hydrophobic interaction with the unsubstituted phenyl ring of **7f**. Overall, the binding affinities of these compounds are comparable to that of the co-crystallized adamantyl derivative AGB.

## Materials and methods

### General details

Melting points (℃, uncorrected) were determined in open glass capillaries utilizing a Stuart SMP30 electro-thermal melting point equipment. The reactions were monitored, and the purity of the end products was assessed using thin layer chromatography (TLC) on silica gel pre-coated aluminum sheets (60 F_254_, Merck), employing petroleum ether/ethyl acetate (7:3) as the eluent and visualizing under ultraviolet light (UV) at 365 and 254 nm. Nuclear magnetic resonance (NMR) spectra were acquired using a Bruker 400 Avance III at 400.20 MHz for ^1^H and 100.63 MHz for ^13^C, employing CDCl_3_ or DMSO-*d*_*6*_ as solvents. Chemical shifts are denoted in *δ* (ppm) downfield from tetramethylsilane (TMS) as the internal standard; and coupling constants (*J*) are reported in Hz. Elemental analyses (C, H, N, and S) were in agreement with the proposed structures within ± 0.3% of the theoretical values. All chemicals and solvents were purchased from commercial suppliers and used without additional purification. The reference drugs Ampicillin trihydrate (CAS # 7177-48-2) and Ciprofloxacin (CAS # 85721-33-1), Fluconazole (CAS # 86386-73-4), Doxorubicin (CAS 23214-92-8) and Sorafenib (CAS # 284461-73-0) were purchased from Sigma-Aldrich Chemie GmbH (Germany).

### General procedure for the synthesis of 2-(adamantan-2-ylidene)-*N*-substituted hydrazine-1-carbothioamides 5a-d

2-Adamantanone **4** (0.3 gm, 0.02 mol) was added to a solution of the appropriate *N*-substituted hydrazinecarbothioamide **3a-d** (0.02 mol) in ethanol (10 mL) containing acetic acid (2 mL), was heated at reflux temperature for 6 h; and then placed aside at room temperature for 2 h. The precipitated crude products were collected, washed with cold ethanol and recrystallized from ethanol or aqueous ethanol (Table 1).

2-(Adamantan-2-ylidene)-*N*-phenylhydrazine-1-carbothioamide **5a**. ^1^H NMR (CDCl_3_): *δ* 9.39 (s, 1H, NH), 8.76 (s, 1H, NH), 7.45 (d, 2 H, Ar-H, *J* = 8.1 Hz), 7.24–7.32 (m, 3 H, Ar-H), 3.13 (s, 1H, Adamantane-H), 2.69 (s, 1H, Adamantane-H), 1.78–2.11 (m, 12 H, Adamantane-H). ^13^C NMR (CDCl_3_): *δ* 176.57 (C = S), 138.56, 126.11, 124.48 (Ar–C), 163.49, 40.01, 38.17, 36.53, 31.91, 28.08 (Adamantane-C).

2-(Adamantan-2-ylidene)-*N*-(4-fluorophenyl)hydrazine-1-carbothioamide **5b**. ^1^H NMR (CDCl_3_) *δ* 9.23 (s, 1H, NH), 8.79 (s, 1H, NH), 7.57 (d, 2 H, Ar-H, *J* = 6.9 Hz), 7.04–7.06 (m, 2 H, Ar–H), 3.73 (s, 1H, Adamantane-H), 3.08 (s, 1H, Adamantane-H), 2.62 (s, 1H, Adamantane-H), 1.94–2.04 (m, 9 H, Adamantane-H), 1.23 (d, 2 H, *J* = 7.0 Hz, Adamantane-H). ^13^C NMR (CDCl_3_): *δ* 176.37 (C = S), 159.07, 133.88, 126.22, 114.82 (Ar–C), 163.32, 39.37, 38.94, 37.55, 35.86, 31.32, 27.42 (Adamantane-C).

2-(Adamantan-2-ylidene)-*N*-(4-chlorophenyl)hydrazine-1-carbothioamide **5c**. ^1^H NMR (CDCl_3_): *δ* 9.30 (s, 1H, NH), 8.74 (s, 1H, NH), 7.61 (d, 2 H, Ar-H, *J* = 8.8 Hz), 7.33 (s, 2 H, Ar–H), 3.06 (s, 1H, Adamantane-H), 2.62 (s, 1H, Adamantane-H), 1.80–2.04 (m, 12 H, Adamantane-H). ^13^C NMR (CDCl_3_): *δ* 175.84 (C = S), 136.49, 130.66, 128.45, 125.30 (Ar–C), 163.35, 38.93, 37.55, 35.84, 31.34, 27.40 (Adamantane-C).

*N*-(Adamantan-1-yl)-2-(adamantan-2-ylidene)hydrazine-1-carbothioamide **5d**. ^1^H NMR (400.20 MHz, CDCl_3_): *δ* 8.15 (s, 1H, NH), 7.48 (s, 1H, NH), 2.97 (s, 1H, Adamantane-H), 2.52 (s, 1H, Adamantane-H), 1.65–2.30 (m, 29 H, Adamantane-H). ^13^C NMR (CDCl_3_): *δ* 175.58 (C = S), 161.48, 54.29, 41.94, 39.88, 39.52, 38.05, 36.69, 31.55, 30.05, 28.12 (Adamantane-C).

### General procedure for the synthesis of (*E*)-2-[(adamantan-2-ylidene)hydrazono]-3,4-disubstituted-2,3-dihydrothiazoles 7a-l

A mixture of the appropriate aryl halomethyl ketone **6a-f** (2.0 mmol), and compound **5a** or **5b** (2.0 mmol), in ethanol (10 mL), was heated under reflux for 24 h. Upon cooling, crushed ice (100 g) and sodium acetate (1.0 g) were added and the mixture was stirred for 30 min and allowed to stand at room temperature for 3 h. The precipitated crude product was filtered, washed with water, dried and crystallized from ethanol or aqueous ethanol (Table 1).

(*E*)-2-[(Adamantan-2-ylidene)hydrazono]-3,4-diphenyl-2,3-dihydrothiazole **7a**. ^1^H NMR (CDCl_3_): *δ* 7.10–7.30 (m, 10 H, Ar–H), 6.09 (s, 1H, Thiazole H), 3.52 (s, 1H, Adamantane-H), 2.73 (s, 1H, Adamantane-H), 1.80–2.02 (m, 12 H, Adamantane-H). ^13^C NMR (CDCl_3_): *δ* 166.29 (Thiazole C-2), 139.95, 138.39, 131.88, 128.10, 126.86 (Ar-C and Thiazole C-4), 100.78 (Thiazole C-5), 172.73, 39.61, 38.34, 37.01, 32.84, 28.28 (Adamantane-C).

(*E*)-[(Adamantan-2-ylidene)hydrazono]-4-(4-fluorophenyl)-3-phenyl-2,3-dihydrothiazole **7b**. ^1^H NMR (DMSO-*d*_*6*_): *δ* 7.05–7.35 (m, 9 H, Ar–H), 6.50 (s, 1H, Thiazole-H), 2.50 (s, 4 H, Adamantane-H), 1.58–1.96 (m, 10 H, Adamantane-H). ^13^C NMR (DMSO-*d*_*6*_): *δ* 167.16 (Thiazole C-2), 163.55, 138.37, 130.94, 130.86, 129.35, 129.10, 128.05, 115.78 (Ar-C and Thiazole C-4), 101.76 (Thiazole C-5), 171.27, 40.56, 40.35, 40.15, 39.94, 39.73, 38.20, 28.13 (Adamantane-C).

(*E*)-[(Adamantan-2-ylidene)hydrazono]-4-(4-chlorophenyl)-3-phenyl-2,3-dihydrothiazole **7c**. ^1^H NMR (DMSO-*d*_*6*_): *δ* 7.09–7.33 (m, 9 H, Ar–H), 6.53 (s, 1H, Thiazole-H), 2.47 (s, 3 H, Adamantane-H), 1.55–1.93 (m, 11 H, Adamantane-H). ^13^C NMR (DMSO-*d*_*6*_): *δ* 167.0 (Thiazole C-2), 138.42, 138.35, 133.30, 130.53, 130.18, 129.26, 128.87, 128.82, 127.96 (Ar-C and Thiazole C-4), 102.43 (Thiazole C-5), 171.27, 40.47, 40.30, 40.13, 39.97, 39.80, 39.63, 39.47, 39.33, 39.19, 38.05, 27.96 (Adamantane-C).

(*E*)-[(Adamantan-2-ylidene)hydrazono]-4-(4-bromophenyl)-3-phenyl-2,3-dihydrothiazole **7d**. ^1^H NMR (DMSO-*d*_*6*_): *δ* 7.04–7.42 (m, 9 H, Ar–H), 6.56 (s, 1H, Thiazole-H), 2.48 (s, 5 H, Adamantane-H), 1.56–1.94 (m, 9 H, Adamantane-H). ^13^C NMR (DMSO-*d*_*6*_): *δ* 167.01 (Thiazole C-2), 138.58, 130.78, 129.20, 128.93, 115.84 (Ar-C and Thiazole C-4), 101.59 (Thiazole C-5), 171.16, 40.57, 40.36, 40.15, 39.73, 39.31, 27.95 (Adamantane-C).

(*E*)-2-[(Adamantan-2-ylidene)hydrazono]-3-phenyl-4-(*p*-tolyl)-2,3-dihydrothiazole **7e**. ^1^H NMR (CDCl_3_): *δ* 7.47–7.57 (m, 9 H, Ar–H), 6.29 (s, 1H, Thiazole-H), 3.77 (s, 1H, Adamantane-H), 2.98 (s, 1H, Adamantane-H), 2.55 (s, 3 H, CH_3_), 1.90–2.25 (m, 12 H, Adamantane-H). ^13^C NMR (CDCl_3_): *δ* 166.05 (Thiazole C-2), 139.72, 137.73, 128.66, 128.20, 128.15, 127.72, 126.54 (Ar-C and Thiazole C-4), 99.78 (Thiazole C-5), 172.36, 39.32, 39.13, 38.05, 36.73, 32.54, 28.0 (Adamantane-C), 21.0 (CH_3_).

(*E*)-2-[(Adamantan-2-ylidene)hydrazono]-4-(4-methoxyphenyl)-3-phenyl-2,3-dihydrothiazole **7f**. ^1^H NMR (CDCl_3_): *δ* 7.14–7.24 (m, 5 H, Ar-H), 6.97 (d, 2 H, Ar-H, *J* = 8.8 Hz), 6.66 (d, 2 H, Ar-H, *J* = 8.0 Hz), 6.29 (s, 1H, Thiazole-H), 3.71 (s, 3 H, OCH_3_), 3.46 (s, 1H, Adamantane-H), 2.67 (s, 1H, Adamantane-H), 1.60–1.97 (m, 12 H, Adamantane-H). ^13^C NMR (CDCl_3_): *δ* 166.32 (Thiazole C-2), 159.42, 139.73, 138.48, 129.51, 128.47, 126.85, 124.41, 113.69 (Ar-C and Thiazole C-4), 99.41 (Thiazole C-5), 55.30 (OCH_3_), 172.60, 39.44, 38.36, 37.04, 32.83, 28.31 (Adamantane-C).

(*E*)-2-[(Adamantan-2-ylidene)hydrazono]-3-(4-fluorophenyl)-4-phenyl-2,3-dihydrothiazole **7 g**. ^1^H NMR (CDCl_3_): *δ* 6.93–7.23 (m, 9 H, Ar–H), 6.09 (s, 1H, Thiazole H), 3.45 (s, 1H, Adamantane-H), 2.66 (s, 1H, Adamantane-H), 1.77–2.01 (m, 12 H, Adamantane-H). ^13^C NMR (CDCl_3_): *δ* 166.54 (Thiazole C-2), 162.45, 159.99, 139.91, 134.10, 131.41, 130.14, 130.05, 128.51, 128.27, 115.69, 115.46 (Ar-C and Thiazole C-4), 100.82 (Thiazole C-5), 166.54, 39.66, 39.38, 39.12, 38.44, 36.88, 32.92, 28.16 (Adamantane-C).

(*E*)-2-[(Adamantan-2-ylidene)hydrazono]-3,4-bis(4-fluorophenyl)-2,3-dihydrothiazole **7 h**. ^1^H NMR (DMSO-*d*_*6*_): *δ* 7.04–7.24 (m, 8 H, Ar–H), 6.46 (s, 1H, Thiazole H), 2.47 (d, 2 H, Adamantane-H, *J* = 6.8 Hz), 1.54–1.92 (m, 12 H, Adamantane-H). ^13^C NMR (DMSO-*d*_*6*_): *δ* 167.09 (Thiazole C-2), 163.22, 162.05, 161.27, 160.11, 138.47, 134.65, 131.15, 131.08, 130.94, 130.87, 128.01, 116.21, 115.91, 115.74 (Ar-C and Thiazole C-4), 101.53 (Thiazole C-5), 171.20, 40.50, 40.33, 40.17, 40.00, 39.83, 39.67, 39.50, 39.34, 38.05, 32.30, 27.97 (Adamantane-C).

(*E*)-2-[(Adamantan-2-ylidene)hydrazono]-4-(4-chlorophenyl)-3-(4-fluorophenyl)-2,3-dihydrothiazole **7i**. ^1^H NMR (CDCl_3_): *δ* 7.13–7.19 (m, 4 H, Ar–H), 6.96–7.03 (m, 4 H, Ar-H), 6.09 (s, 1H, Thiazole H), 3.42 (s, 1H, Adamantane-H), 2.81 (s, 1H, Adamantane-H), 1.76–2.01 (m, 12 H, Adamantane-H). ^13^C NMR (CDCl_3_): *δ* 166.40 (Thiazole C-2), 162.99, 160.13, 138.78, 134.50, 130.65, 130.12, 128.77, 116.16 (Ar-C and Thiazole C-4), 101.45 (Thiazole C-5), 166.40, 39.68, 39.38, 38.93, 38.48, 36.80, 32.99, 28.09 (Adamantane-C).

(*E*)-2-[(Adamantan-2-ylidene)hydrazono]-4-(4-bromophenyl)-3-(4-fluorophenyl)-2,3-dihydrothiazole **7j**. ^1^H NMR (DMSO-*d*_*6*_): *δ* 7.40 (d, 2 H, Ar-H, *J* = 8.4 Hz), 7.02–7.24 (m, 6 H, Ar-H), 6.52 (s, 1H, Thiazole H), 2.45 (s, 1H, Adamantane-H), 1.53–1.91 (m, 12 H, Adamantane-H). ^13^C NMR (DMSO-*d*_*6*_): *δ* 167.04 (Thiazole C-2), 162.06, 160.12, 138.37, 134.57, 131.80, 131.72, 130.54, 122.10, 116.27, 116.09 (Ar-C and Thiazole C-4), 102.36 (Thiazole C-5), 171.39, 40.48, 39.81, 39.64, 39.33, 36.58, 32.34, 27.96 (Adamantane-C).

(*E*)-2-[(Adamantan-2-ylidene)hydrazono]-3-(4-fluorophenyl)-4-(*p*-tolyl)-2,3-dihydrothiazole **7k**. ^1^H NMR (CDCl_3_): *δ* 7.17–7.25 (m, 3 H, Ar-H), 6.97–7.03 (m, 5 H, Ar-H), 6.04 (s, 1H, Thiazole H), 3.47 (s, 1H, Adamantane-H), 2.75 (s, 1H, Adamantane-H), 2.30 (s, 3 H, CH_3_), 1.78–2.02 (m, 12 H, Adamantane-H). ^13^C NMR (CDCl_3_): *δ* 166.21 (Thiazole C-2), 162.07, 159.62, 157.82, 139.84, 138.05, 130.37, 129.85, 128.80, 127.95, 127.81, 122.79, 115.98, 115.91, 115.31, 115.08 (Ar-C and Thiazole C-4), 99.80 (Thiazole C-5), 166.21, 39.32, 38.08, 36.60, 32.56, 27.88, (Adamantane-C), 21.04 (CH_3_).

(*E*)-2-[(Adamantan-2-ylidene)hydrazono]-3-(4-fluorophenyl)-4-(4-methoxyphenyl)-2,3-dihydrothiazole **7 L**. ^1^H NMR (CDCl_3_): *δ* 7.14–7.18 (m, 2 H, Ar-H), 6.71-7.0 (m, 4 H, Ar-H), 6.0 (s, 1H, Thiazole H), 3.75 (s, 3 H, OCH_3_), 3.43 (s, 1H, Adamantane-H), 1.77–2.01 (m, 13H, Adamantane-H). ^13^C NMR (CDCl_3_): *δ* 167.04 (Thiazole C-2), 162.51, 159.65, 139.77, 134.03, 130.17, 129.70, 132.72, 115.52, 113.85 (Ar-C and Thiazole C-4), 99.54 (Thiazole C-5), 55.36 (OCH_3_), 166.28, 39.68, 39.39, 38.47, 36.82, 32.92, 28.12 (Adamantane-C).

### Single-crystal X-ray diffraction

Analytically-pure samples of compounds **5c**,** 7a** and **7f** (40 mg) were dissolved in 10 mL of ethanol: chloroform (1:1, *v/v*), and the solution was allowed to slowly evaporate at room temperature for 48 h.

X-ray intensity data were collected at 160.0(1) K on a Rigaku OD Supernova/Atlas diffractometer using Cu Kα radiation (λ = 1.54184 Å) from a dual wavelength X-ray source and an Oxford Instruments Cryojet XL cooler. A suitable single crystal was selected, mounted using polybutene oil on a flexible loop fixed on a goniometer head, and immediately transferred to the diffractometer. Pre-experiment, data collection, data reduction and analytical absorption correction [56] were performed with the program suite CrysAlisPro (ver. 1.171.41.113a, Rigaku Oxford Diffraction, 2019). Using *Olex2* [57], the structures were solved with the SHELXT small molecule structure solution program [58], and refined with the SHELXL 2018/3 package [59] by full-matrix least-squares minimization on F^2^. The PLATON program was used to check the results of the X-ray analysis [60]. Crystal packing and molecular dimers were produced using the MERCURY program [61]. Compound **5c** co-crystallized with water molecule in a 1:0.06 ratio. The water oxygen atom lies on a two-fold axis. The O–H bond length of the water molecule was restrained to 0.82(1) Å. The H atoms bound to nitrogen atoms were located from a difference Fourier map and refined freely along with their isotropic displacement parameters. In all three compounds, hydrogen atoms bound to carbon atoms were placed in geometrically calculated positions. PLATON [60] was used to check the results of the X-ray analysis.

### Molecular docking analysis

All molecular docking simulations were performed using the CB-Dock2 web server [62], which utilizes AutoDock Vina for scoring [63]. The crystal structure of dehydrosqualene synthase (CrtM) from *Staphylococcus aureus*, complexed with an adamantyl derivative (ligand ID: RWZ), was retrieved from the Protein Data Bank (PDB ID: 4EA2). Similarly, the crystal structure of human urokinase-type plasminogen activator complexed with *N*-(1-adamantyl)-*N*’-(4-guanidinobenzyl)urea (ligand ID: AGB) was obtained from the PDB (ID: 1EJN). The most potent antibacterial compounds (**7f** and **7i**) and the most effective anti-proliferative agents (**5a**, **5b** and **7f**) were subjected to molecular docking against these targets to evaluate their binding affinities and binding modes at the active sites. The protein-ligand interactions were analyzed using the PLIP web server [64] based on the predicted poses.

## Conclusions

This work focused on synthesizing adamantane-thiazole hybrid molecules from the antecedents 2-(adamantan-2-ylidene)-*N*-substituted hydrazine-1-carbothioamides. Structural verification by X-ray crystallography confirmed the compounds to be 2-[(adamantan-2-ylidene)hydrazono]-3,4-disubstituted-2,3-dihydrothiazole, rather than their isomeric forms. Antimicrobial evaluation revealed that compound **7i**, with two halophenyl substituents, exhibited significant activity against Gram-positive (*Staphylococcus aureus and Bacillus subtilis*) and Gram-negative (*Escherichia coli*) bacteria, but no antifungal activity was displayed by the tested compounds. In cytotoxicity assays (MTT test), most thiazole compounds showed weak anti-proliferative effects, except for the 4-methoxyphenyl analogues **7f** and **7 L**. It is important to note that adamantane derivatives with thiosemicarbazone side chains (**5a**, **5b**,** 5c**) were among the most active antibacterial derivatives, and had the strongest anti-proliferative activity. Molecular docking studies of the active adamantane-thiazole derivatives **7f** and **7i** against bacteria targets demonstrated strong affinity for *Staphylococcus aureus* dehydrosqualene synthase (*Sa*CrtM). Significant affinity for the urokinase-type plasminogen activator receptor (uPAR) was also displayed by the anti-proliferative active molecules **5a**, **5b**, and **7f**.

## Supplementary Information

Below is the link to the electronic supplementary material.

Supplementary material 1.

## Data Availability

The crystallographic data for the structures of compounds **5c** (CCDC #: 2477371), **7a** (CCDC #: 2477374), and **7f** (CCDC #: 2477375) could be obtained free of charge from the Cambridge Crystallographic Data Centre (www.ccdc.cam.ac.uk/data\_request/cif).
