# Supplementary material for: Adamantane-thiazole hybrids and related derivatives: synthesis, crystal structures, in vitro antibacterial, antifungal, and anti-proliferative activities
Source: BMC Chem. 2026 Jan 20;20(1):26. doi: 10.1186/s13065-025-01706-9 (PMC12905878; doi:10.1186/s13065-025-01706-9)
Supplement: Supplementary file 1 — Supplementary material 1. [file 13065_2025_1706_MOESM1_ESM.pdf]

Supporting Information

Adamantane-thiazole hybrids and related derivatives. Synthesis, crystal structures, *in vitro* antibacterial, antifungal, and anti-proliferative activities

Heidi S. Abd El-Monaem, Mahmoud B. El-Ashmawy, Naglaa I. Abdel-Aziz, Olivier Blacque, El-Sayed E. Habib, Subbiah Thamotharan and Ali A. El-Emam

Contents

1. Molecular formulae, molecular weights and elemental analyses data of compounds **5a-d** and **7a-l** (Table S1).
2. CheckCif reports of compounds **5c**, **7a** and **7f**.
3. Determination of *in vitro* antimicrobial activity for compounds **5a-d** and **7a-l** (agar disc-diffusion method).
4. Determination of minimal inhibitory concentrations (MIC) of compounds **5a**, **5b**, **5c**, **7a**, **7i** and **7k** (micro-dilution susceptibility method).
5. Determination of *in vitro* anti-proliferative activity of compounds **5a-d** and **7a-l** (MTT assay).
6. ^{1}H NMR and ^{13}C NMR spectra.

Table S1. Molecular formulae, molecular weights and elemental analyses data of compounds **5a-d** and **7b-**

Comp. No.	Mol. Formula (Mol. Wt.)	Analysis: % Calcd. (Found)			
		C	H	N	S
**5a**	$C_{17}H_{21}N_3S$ (299.44)	68.19 (68.13)	7.07 (7.10)	14.03 (14.0)	10.71 (10.68)
**5b**	$C_{17}H_{20}FN_3S$ (317.43)	64.33 (64.28)	6.35 (6.36)	13.24 (13.22)	10.10 (10.10)
**5c**	$C_{17}H_{20}ClN_3S$ (333.88)	61.16 (61.10)	6.04 (6.10)	12.59 (12.54)	9.60 (9.56)
**5d**	$C_{21}H_{31}N_3S$ (357.56)	70.54 (70.51)	8.74 (8.77)	11.75 (11.73)	8.97 (8.95)
**7a**	$C_{25}H_{25}N_3S$ (399.56)	75.15 (75.10)	6.31 (6.33)	10.52 (10.44)	8.02 (8.01)
**7b**	$C_{25}H_{24}FN_3S$ (417.55)	71.91 (71.88)	5.79 (5.81)	10.06 (10.02)	7.68 (7.66)
**7c**	$C_{25}H_{24}ClN_3S$ (434.0)	69.19 (69.13)	5.57 (5.60)	9.68 (9.56)	7.39 (7.38)
**7d**	$C_{25}H_{24}BrN_3S$ (478.45)	62.76 (62.72)	5.06 (5.12)	8.78 (8.74)	6.70 (6.65)
**7e**	$C_{26}H_{27}N_3S$ (413.58)	75.51 (75.44)	6.58 (6.60)	10.16 (10.12)	7.75 (7.74)
**7f**	$C_{26}H_{27}N_3OS$ (429.58)	72.70 (72.68)	6.34 (6.36)	9.78 (9.75)	7.46 (7.46)
**7g**	$C_{25}H_{24}FN_3S$ (417.55)	71.91 (71.84)	5.79 (5.83)	10.06 (10.03)	7.68 (7.67)
**7h**	$C_{25}H_{23}F_2N_3S$ (435.54)	68.94 (68.90)	5.32 (5.36)	9.65 (9.64)	7.36 (7.37)
**7i**	$C_{25}H_{23}ClFN_3S$ (451.99)	66.43 (66.32)	5.13 (5.15)	9.30 (9.27)	7.09 (7.10)
**7j**	$C_{25}H_{23}BrFN_3S$ (496.44)	60.49 (60.25)	4.67 (4.70)	8.46 (8.38)	6.46 (6.43)
**7k**	$C_{26}H_{26}FN_3S$ (431.57)	72.36 (72.29)	6.07 (6.11)	9.74 (9.75)	7.43 (7.41)
**7l**	$C_{26}H_{26}FN_3OS$ (447.57)	69.77 (69.68)	5.86 (5.90)	9.39 (9.42)	7.16 (7.15)

checkCIF/PLATON report

Structure factors have been supplied for datablock(s) emam2404

THIS REPORT IS FOR GUIDANCE ONLY. IF USED AS PART OF A REVIEW PROCEDURE FOR PUBLICATION, IT SHOULD NOT REPLACE THE EXPERTISE OF AN EXPERIENCED CRYSTALLOGRAPHIC REFEREE.

No syntax errors found. CIF dictionary Interpreting this report

Datablock: emam2404

Bond precision: C-C = 0.0024 A Wavelength=1.54184

Cell:	a=24.1495(3)	b=6.4705(1)	c=21.2674(3)
	alpha=90	beta=102.890(1)	gamma=90
Temperature:	160 K		

	Calculated	Reported
Volume	3239.49(8)	3239.49(8)
Space group	C 2/c	C 1 2/c 1
Hall group	-C 2yc	-C 2yc
Moiety formula	C17 H20 Cl N3 S, 0.058(H2 O)	C17 H20 Cl N3 S, 0.058(H2 O)
Sum formula	C17 H20.12 Cl N3 O0.06 S	C17 H20.12 Cl N3 O0.06 S
Mr	334.92	334.93
Dx,g cm-3	1.373	1.373
Z	8	8
Mu (mm-1)	3.282	3.282
F000	1412.7	1413.0
F000'	1421.29	
h,k,lmax	30,8,26	30,8,26
Nref	3397	3374
Tmin,Tmax	0.538,0.653	0.611,0.734
Tmin'	0.448	

Correction method= # Reported T Limits: Tmin=0.611 Tmax=0.734
AbsCorr = ANALYTICAL

Data completeness= 0.993 Theta(max)= 76.210

R(reflections)= 0.0354(3127) wR2(reflections)= 0.0942(3374)

S = 1.065 Npar= 213

The following ALERTS were generated. Each ALERT has the format
test-name_ALERT_alert-type_alert-level.
Click on the hyperlinks for more details of the test.

Alert level C

```
PLAT077_ALERT_4_C Unitcell Contains Non-integer Number of Atoms ..     Please Check
```

Alert level G

```
PLAT002_ALERT_2_G Number of Distance or Angle Restraints on AtSite          2 Note
PLAT007_ALERT_5_G Number of Unrefined Donor-H Atoms .............          1 Report
              H1A
PLAT068_ALERT_1_G Reported F000 Differs from Calcd (or Missing)...     Please Check
PLAT172_ALERT_4_G The CIF-Embedded .res File Contains DFIX Records          1 Report
PLAT173_ALERT_4_G The CIF-Embedded .res File Contains DANG Records          1 Report
PLAT302_ALERT_4_G Anion/Solvent/Minor-Residue Disorder (Resd    2)       100% Note
PLAT304_ALERT_4_G Non-Integer Number of Atoms in ..... (Resd    2)       0.35 Check
PLAT860_ALERT_3_G Number of Least-Squares Restraints ............          2 Note
PLAT912_ALERT_4_G Missing # of FCF Reflections Above STh/L=  0.600         22 Note
PLAT969_ALERT_5_G The 'Henn et al.' R-Factor-gap value ..........       3.60 Note
              Predicted wR2: Based on SigI**2  2.62 or SHELX Weight  9.13
PLAT978_ALERT_2_G Number C-C Bonds with Positive Residual Density.         14 Info
```

   0 **ALERT level A** = Most likely a serious problem - resolve or explain
   0 **ALERT level B** = A potentially serious problem, consider carefully
   1 **ALERT level C** = Check. Ensure it is not caused by an omission or oversight
  11 **ALERT level G** = General information/check it is not something unexpected

```
   1 ALERT type 1 CIF construction/syntax error, inconsistent or missing data
   2 ALERT type 2 Indicator that the structure model may be wrong or deficient
   1 ALERT type 3 Indicator that the structure quality may be low
   6 ALERT type 4 Improvement, methodology, query or suggestion
   2 ALERT type 5 Informative message, check
```

It is advisable to attempt to resolve as many as possible of the alerts in all categories. Often the minor alerts point to easily fixed oversights, errors and omissions in your CIF or refinement strategy, so attention to these fine details can be worthwhile. In order to resolve some of the more serious problems it may be necessary to carry out additional measurements or structure refinements. However, the purpose of your study may justify the reported deviations and the more serious of these should normally be commented upon in the discussion or experimental section of a paper or in the "special_details" fields of the CIF. checkCIF was carefully designed to identify outliers and unusual parameters, but every test has its limitations and alerts that are not important in a particular case may appear. Conversely, the absence of alerts does not guarantee there are no aspects of the results needing attention. It is up to the individual to critically assess their own results and, if necessary, seek expert advice.

Publication of your CIF in IUCr journals

A basic structural check has been run on your CIF. These basic checks will be run on all CIFs submitted for publication in IUCr journals (*Acta Crystallographica*, *Journal of Applied Crystallography*, *Journal of Synchrotron Radiation*); however, if you intend to submit to *Acta Crystallographica Section C* or *E* or *IUCrData*, you should make sure that full publication checks are run on the final version of your CIF prior to submission.

Publication of your CIF in other journals

Please refer to the *Notes for Authors* of the relevant journal for any special instructions relating to CIF submission.

PLATON version of 06/01/2024; check.def file version of 05/01/2024

Datablock emam2404 - ellipsoid plot

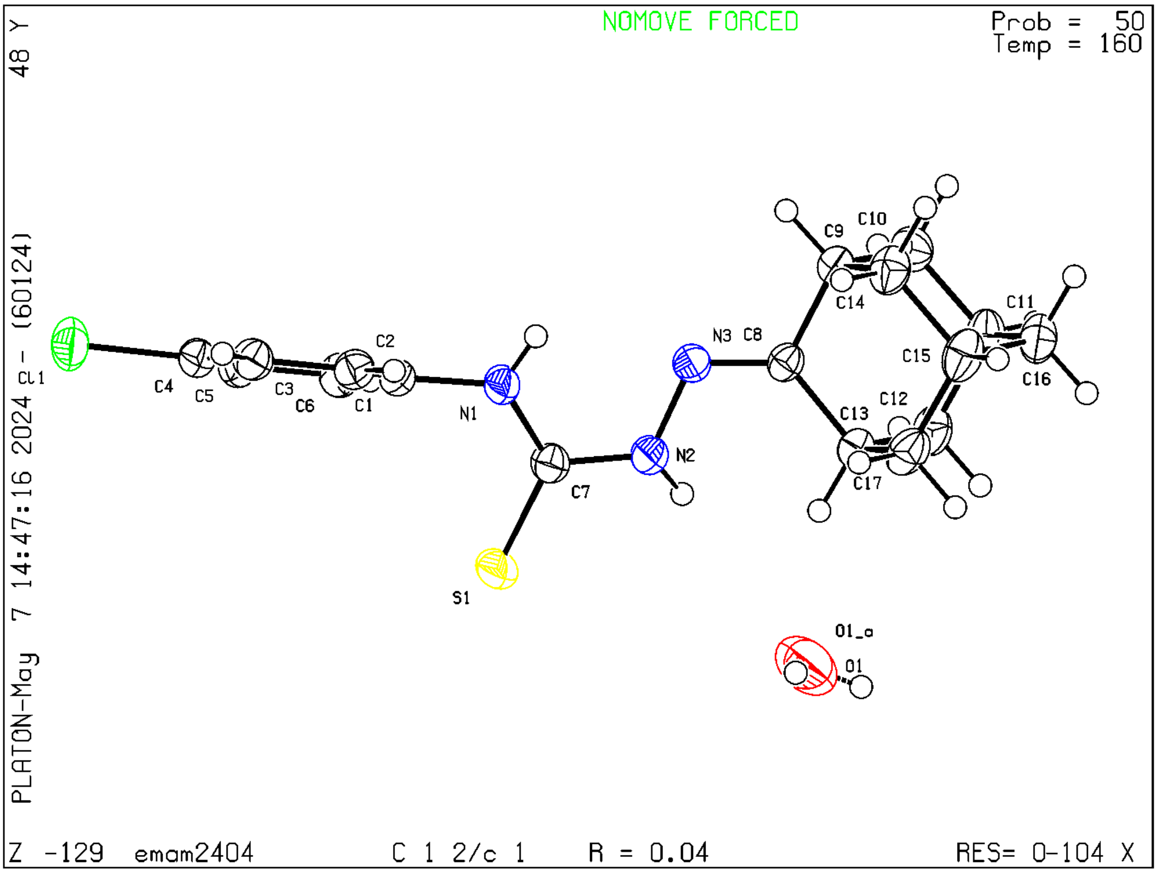

checkCIF/PLATON report

Structure factors have been supplied for datablock(s) emam2111

THIS REPORT IS FOR GUIDANCE ONLY. IF USED AS PART OF A REVIEW PROCEDURE FOR PUBLICATION, IT SHOULD NOT REPLACE THE EXPERTISE OF AN EXPERIENCED CRYSTALLOGRAPHIC REFEREE.

No syntax errors found. CIF dictionary Interpreting this report

Datablock: emam2111

```
Bond precision:   C-C = 0.0029 A          Wavelength=1.54184

Cell:            a=10.82409(12)     b=6.51732(9)       c=30.0707(3)
                 alpha=90           beta=96.7639(9)    gamma=90
Temperature:     160 K

                 Calculated                   Reported
Volume           2106.55(4)                   2106.55(4)
Space group      P 21/c                       P 1 21/c 1
Hall group       -P 2ybc                      -P 2ybc
Moiety formula   C25 H25 N3 S                 C25 H25 N3 S
Sum formula      C25 H25 N3 S                 C25 H25 N3 S
Mr               399.54                       399.54
Dx,g cm-3        1.260                        1.260
Z                4                            4
Mu (mm-1)        1.472                        1.472
F000             848.0                        848.0
F000'            851.37
h,k,lmax         13,8,37                      13,8,37
Nref             4313                         4288
Tmin,Tmax        0.838,0.957                  0.745,0.874
Tmin'            0.734

Correction method= # Reported T Limits: Tmin=0.745 Tmax=0.874
AbsCorr = ANALYTICAL

Data completeness= 0.994          Theta(max)= 74.476

R(reflections)= 0.0433( 3880)                   wR2(reflections)=
                                                0.1155( 4288)

S = 1.061                  Npar= 263
```

The following ALERTS were generated. Each ALERT has the format
test-name_ALERT_alert-type_alert-level.
Click on the hyperlinks for more details of the test.

Alert level C

```
PLAT230_ALERT_2_C Hirshfeld Test Diff for    C1       --C6       .        6.0 s.u.
PLAT906_ALERT_3_C Large K Value in the Analysis of Variance ......      2.047 Check
PLAT911_ALERT_3_C Missing FCF Refl Between Thmin & STh/L=    0.600         17 Report
               -3  5  2,   4  7  2,  -3  5  3, -11  1  5,   0  7  8,   1  7  8,
               11  3  8,   1  7  9,  11  3  9,   6  6 10,  10  3 10,   1  7 12,
                1  7 13, -10  4 16,   9  3 17,  -1  5 22,  -1  5 23,
PLAT918_ALERT_3_C Reflection(s) with I(obs) much Smaller I(calc) .          1 Check
```

Alert level G

```
PLAT143_ALERT_4_G s.u. on c - Axis Small or Missing .............    0.00030 Ang.
PLAT912_ALERT_4_G Missing # of FCF Reflections Above STh/L=  0.600          8 Note
PLAT933_ALERT_2_G Number of HKL-OMIT Records in Embedded .res File          7 Note
              -11  1  5,   1  7  8,   4  7  2,   6  6 10,   8  6  6,   9  3 17,
               10  3 10,
PLAT969_ALERT_5_G The 'Henn et al.' R-Factor-gap value ..........      5.542 Note
              Predicted wR2: Based on SigI**2  2.08 or SHELX Weight 10.88
PLAT978_ALERT_2_G Number C-C Bonds with Positive Residual Density.          8 Info
```

0 **ALERT level A** = Most likely a serious problem - resolve or explain
0 **ALERT level B** = A potentially serious problem, consider carefully
4 **ALERT level C** = Check. Ensure it is not caused by an omission or oversight
5 **ALERT level G** = General information/check it is not something unexpected

0 ALERT type 1 CIF construction/syntax error, inconsistent or missing data
3 ALERT type 2 Indicator that the structure model may be wrong or deficient
3 ALERT type 3 Indicator that the structure quality may be low
2 ALERT type 4 Improvement, methodology, query or suggestion
1 ALERT type 5 Informative message, check

It is advisable to attempt to resolve as many as possible of the alerts in all categories. Often the minor alerts point to easily fixed oversights, errors and omissions in your CIF or refinement strategy, so attention to these fine details can be worthwhile. In order to resolve some of the more serious problems it may be necessary to carry out additional measurements or structure refinements. However, the purpose of your study may justify the reported deviations and the more serious of these should normally be commented upon in the discussion or experimental section of a paper or in the "special_details" fields of the CIF. checkCIF was carefully designed to identify outliers and unusual parameters, but every test has its limitations and alerts that are not important in a particular case may appear. Conversely, the absence of alerts does not guarantee there are no aspects of the results needing attention. It is up to the individual to critically assess their own results and, if necessary, seek expert advice.

Publication of your CIF in IUCr journals

A basic structural check has been run on your CIF. These basic checks will be run on all CIFs submitted for publication in IUCr journals (*Acta Crystallographica*, *Journal of Applied Crystallography*, *Journal of Synchrotron Radiation*); however, if you intend to submit to *Acta Crystallographica Section C* or *E* or *IUCrData*, you should make sure that full publication checks are run on the final version of your CIF prior to submission.

Publication of your CIF in other journals

Please refer to the *Notes for Authors* of the relevant journal for any special instructions relating to CIF submission.

PLATON version of 11/11/2024; check.def file version of 11/11/2024

Datablock emam2111 - ellipsoid plot

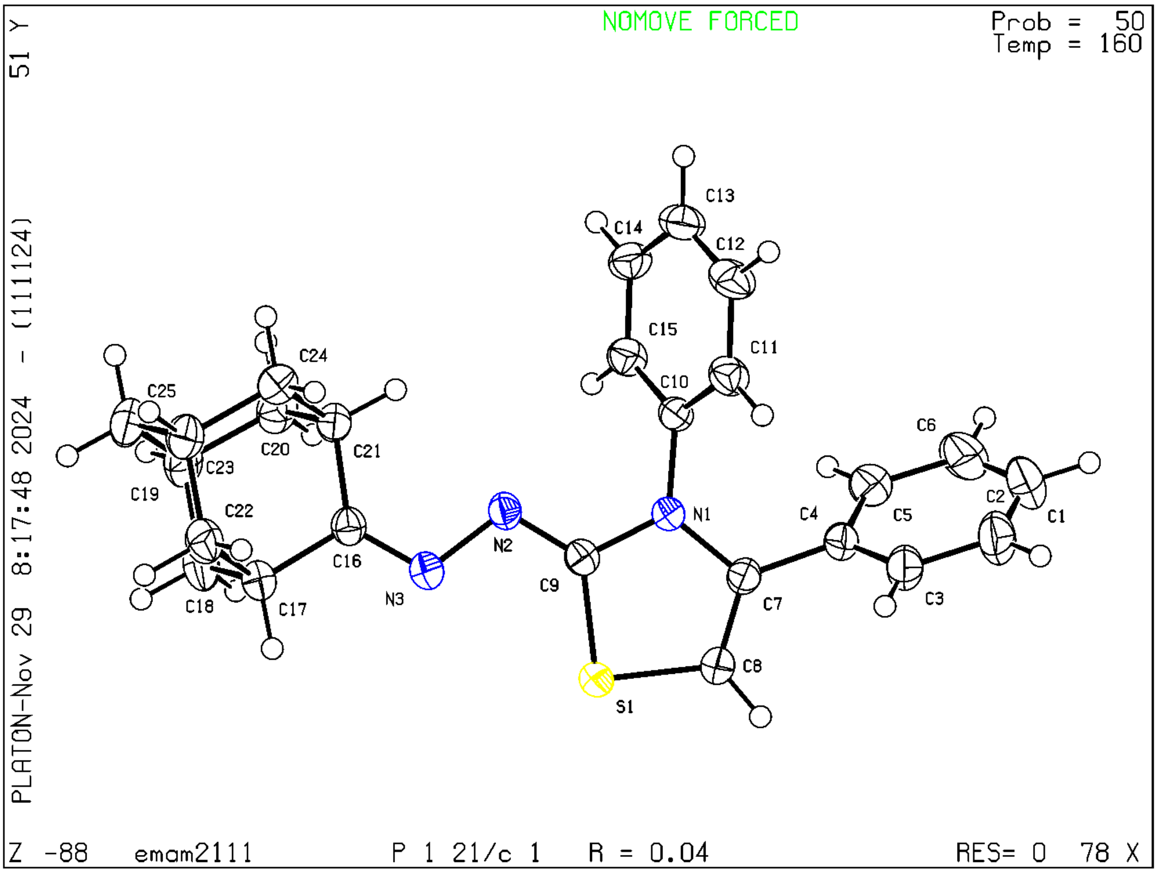

checkCIF/PLATON report

Structure factors have been supplied for datablock(s) emam0705

THIS REPORT IS FOR GUIDANCE ONLY. IF USED AS PART OF A REVIEW PROCEDURE FOR PUBLICATION, IT SHOULD NOT REPLACE THE EXPERTISE OF AN EXPERIENCED CRYSTALLOGRAPHIC REFEREE.

No syntax errors found. CIF dictionary Interpreting this report

Datablock: emam0705

Bond precision:	C-C = 0.0034 A		Wavelength=1.54184
Cell:	a=11.0164(1)	b=6.5217(1)	c=15.6370(2)
	alpha=90	beta=101.434(1)	gamma=90
Temperature:	160 K		

	Calculated	Reported
Volume	1101.15(2)	1101.15(2)
Space group	P 21	P 1 21 1
Hall group	P 2yb	P 2yb
Moiety formula	C26 H27 N3 O S	C26 H27 N3 O S
Sum formula	C26 H27 N3 O S	C26 H27 N3 O S
Mr	429.57	429.56
Dx,g cm-3	1.296	1.296
Z	2	2
Mu (mm-1)	1.480	1.480
F000	456.0	456.0
F000'	457.82	
h,k,lmax	13,8,19	13,8,19
Nref	4492[2452]	4485
Tmin,Tmax	0.823,0.957	0.769,0.963
Tmin'	0.711	

Correction method= # Reported T Limits: Tmin=0.769 Tmax=0.963
AbsCorr = ANALYTICAL

Data completeness= 1.83/1.00 Theta(max)= 74.503

R(reflections)= 0.0342(4259) wR2(reflections)= 0.0881(4485)

S = 1.046 Npar= 281

```
The following ALERTS were generated. Each ALERT has the format
       test-name_ALERT_alert-type_alert-level.
Click on the hyperlinks for more details of the test.
```

Alert level G

```
PLAT969_ALERT_5_G The 'Henn et al.' R-Factor-gap value ..........       3.14 Note
              Predicted wR2: Based on SigI**2  2.81 or SHELX Weight  8.70
PLAT978_ALERT_2_G Number C-C Bonds with Positive Residual Density.         1 Info
```

```
   0 ALERT level A = Most likely a serious problem - resolve or explain
   0 ALERT level B = A potentially serious problem, consider carefully
   0 ALERT level C = Check. Ensure it is not caused by an omission or oversight
   2 ALERT level G = General information/check it is not something unexpected

   0 ALERT type 1 CIF construction/syntax error, inconsistent or missing data
   1 ALERT type 2 Indicator that the structure model may be wrong or deficient
   0 ALERT type 3 Indicator that the structure quality may be low
   0 ALERT type 4 Improvement, methodology, query or suggestion
   1 ALERT type 5 Informative message, check
```

It is advisable to attempt to resolve as many as possible of the alerts in all categories. Often the minor alerts point to easily fixed oversights, errors and omissions in your CIF or refinement strategy, so attention to these fine details can be worthwhile. In order to resolve some of the more serious problems it may be necessary to carry out additional measurements or structure refinements. However, the purpose of your study may justify the reported deviations and the more serious of these should normally be commented upon in the discussion or experimental section of a paper or in the "special_details" fields of the CIF. checkCIF was carefully designed to identify outliers and unusual parameters, but every test has its limitations and alerts that are not important in a particular case may appear. Conversely, the absence of alerts does not guarantee there are no aspects of the results needing attention. It is up to the individual to critically assess their own results and, if necessary, seek expert advice.

Publication of your CIF in IUCr journals

A basic structural check has been run on your CIF. These basic checks will be run on all CIFs submitted for publication in IUCr journals (*Acta Crystallographica*, *Journal of Applied Crystallography*, *Journal of Synchrotron Radiation*); however, if you intend to submit to *Acta Crystallographica Section C* or *E* or *IUCrData*, you should make sure that full publication checks are run on the final version of your CIF prior to submission.

Publication of your CIF in other journals

Please refer to the *Notes for Authors* of the relevant journal for any special instructions relating to CIF submission.

PLATON version of 06/01/2024; check.def file version of 05/01/2024

Datablock emam0705 - ellipsoid plot

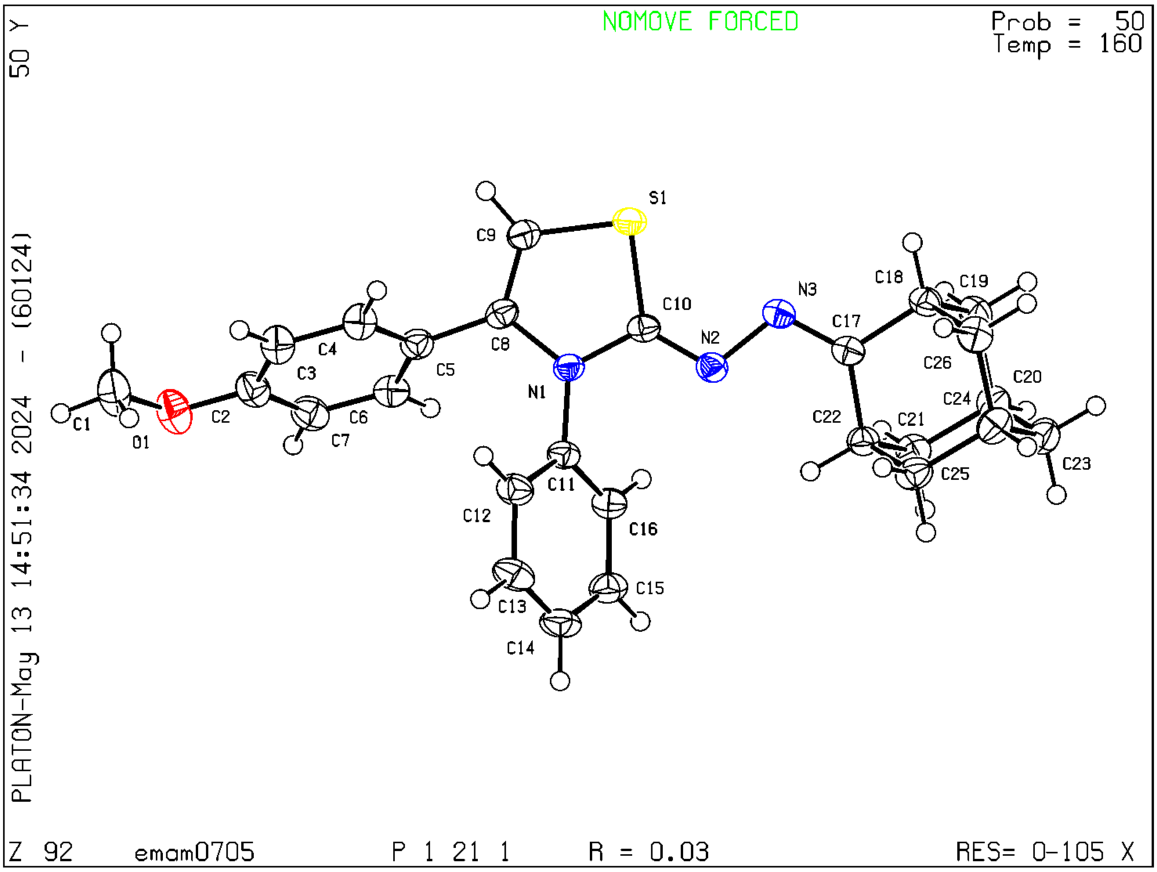

Determination of *in vitro* antimicrobial activity of compounds 5a-d and 7a-l (agar disc-diffusion method)

Sterile filter paper discs (8 mm diameter) were moistened with compounds **5a-d** and **7a-l** solution in dimethyl sulfoxide of specific concentration (200 μg/disc), the broad-spectrum antibacterial drugs Ampicillin trihydrate, Ciprofloxacin and the antifungal drug Fluconazole (100 μg/disc) were carefully placed on the agar culture plates that had been previously inoculated separately with the microorganisms. The plates were incubated at 37 °C, and the diameter of the growth inhibition zones were measured after 24 hours in case of bacteria and 48 hours in case of fungi.

Determination of minimal inhibitory concentrations (MIC) for compounds 5a, 5b, 5c, 7a, 7i and 7k (micro-dilution susceptibility method)

Compounds **5a**, **5b**, **5c**, **7a**, **7i** and **7k**, Ampicillin trihydrate and Ciprofloxacin were dissolved in dimethyl sulfoxide at concentration of 128 μg/mL. Two-fold dilutions of the solution were prepared (128, 64, 32, …, 0.5 μg/mL). The microorganism suspensions at 10^6 CFU/mL (colony forming unit/ml) concentrations were inoculated to the corresponding wells and the plates were incubated at 36 °C for 24 hours. The MIC values were determined as the lowest concentration that completely inhibited visible growth of the microorganism as detected by unaided eye. The MBC values were determined by the lowest concentration that killed of the microorganism by re-cultured on agar medium to verify the absence of growth.

Determination of *in vitro* anti-proliferative activity for compounds 5a-d and 7a-l (MTT assay)

The tumor cells (3000 cells per well) were cultured and seeded into 96-well plates and the plates were incubated for 24 hours. The cells were then treated with compounds **5a-d** and **7a-l**, Doxorubicin and Sorafenib, at different concentrations in dimethyl sulfoxide (0.1 μM to 100 μM) at 37 °C in an atmosphere of 5% CO_2 for 48 hours. Freshly prepared 3-[4,5-dimethylthiazoyl-2-yl]-2,5-diphenyltetrazolium bromide (MTT) was added to each well at a terminal concentration of 5 μg/mL and incubated with cells at 37 °C for 4 hours. The formazan crystals were dissolved in 100 μL of dimethyl sulfoxide in each well, and the absorbency at 492 nm (for absorbance of MTT formazan) and 630 nm (for the reference wavelength) was measured with an enzyme linked

immunosorbent assay (ELISA) reader (ChroMate-4300, FL, USA). All compounds were tested three times in each of the cell lines. The IC_{50} values were calculated according to the equation for Boltzmann sigmoidal concentration response curve using the nonlinear regression fitting models (Graph Pad, Prism Version 5). The results reported are means of three separate experiments. Statistical differences were analyzed according to one-way ANOVA test wherein the differences were considered to be significant at $p < 0.05$.

^{1}H NMR and ^{13}C NMR spectra

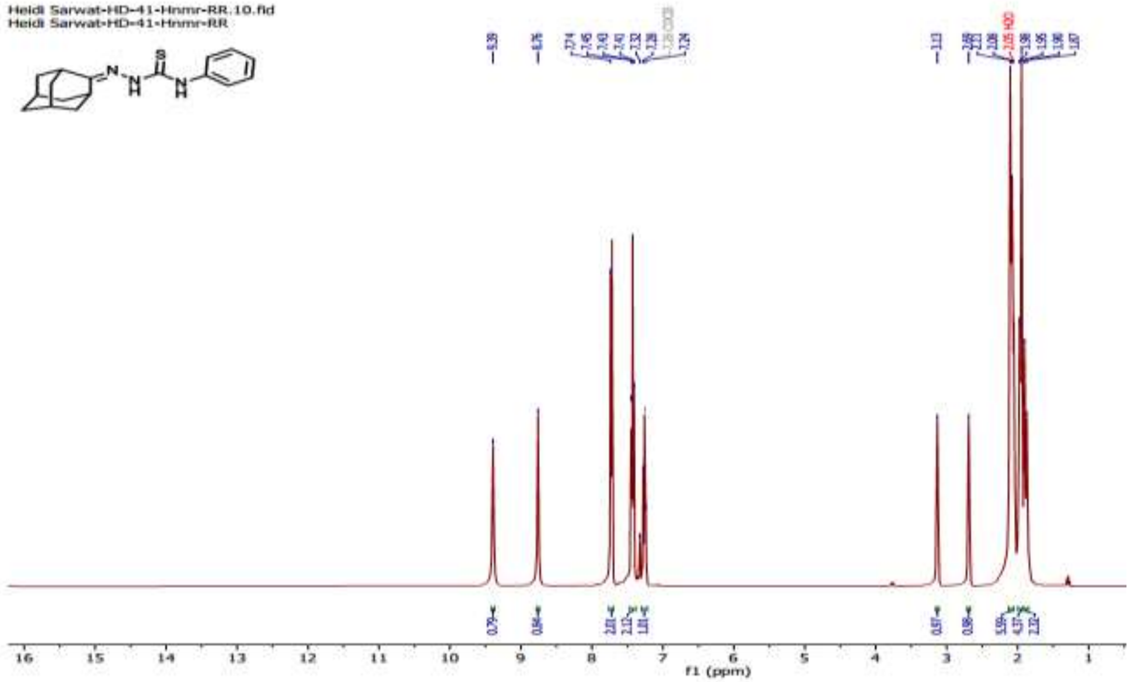

^{1}H NMR (400.16 MHz) of compound **5a**

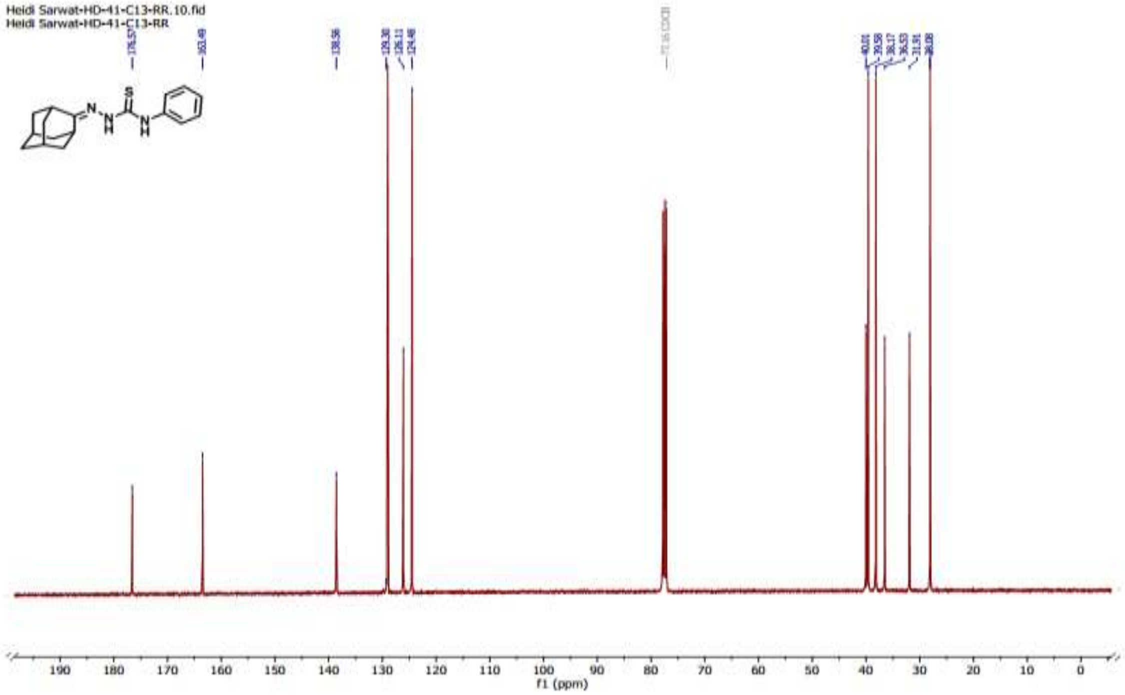

^{13}C NMR (100.63 MHz) of compound **5a**

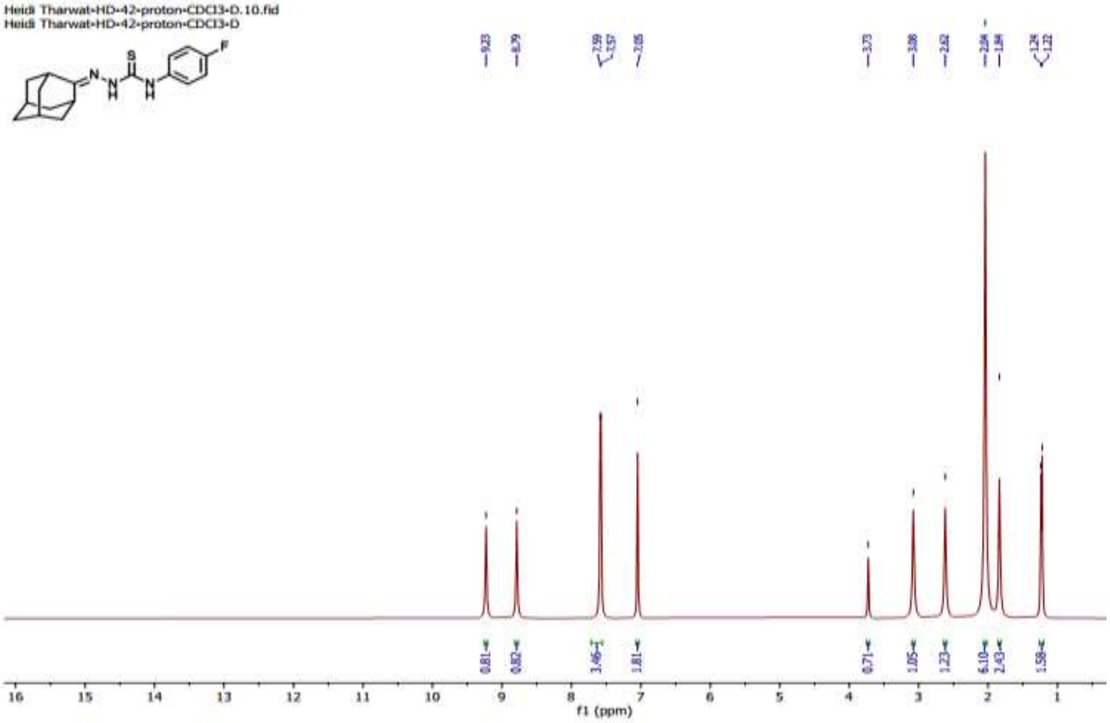

^{1}H NMR (400.20 MHz) of compound **5b**

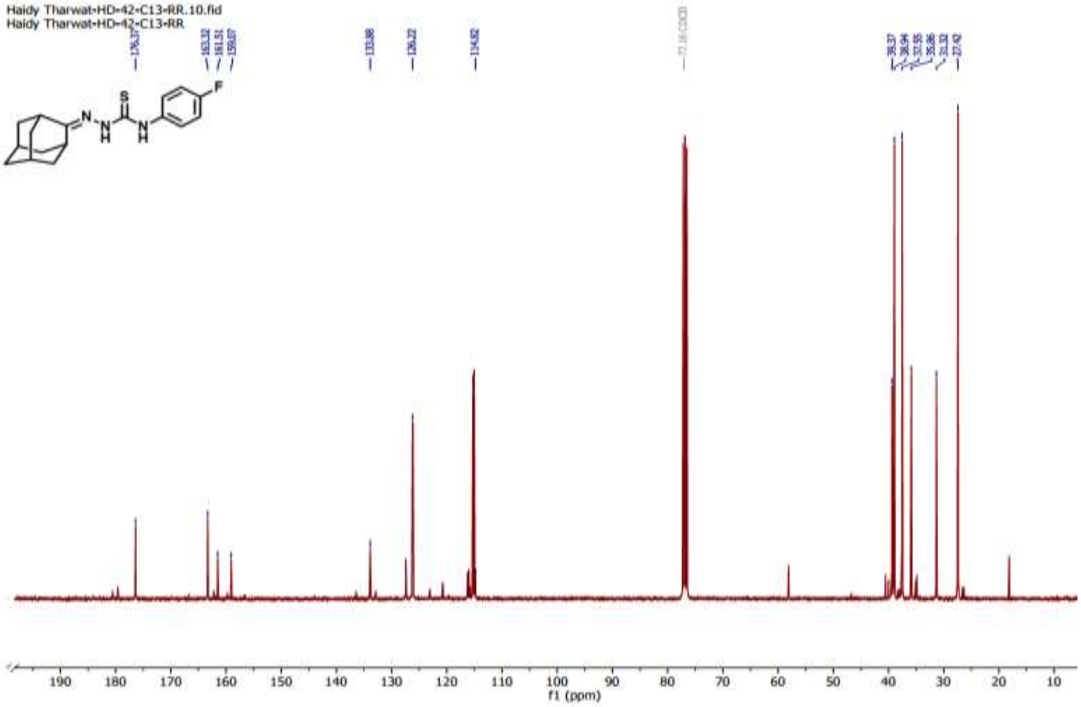

^{13}C NMR (100.63 MHz) of compound **5b**

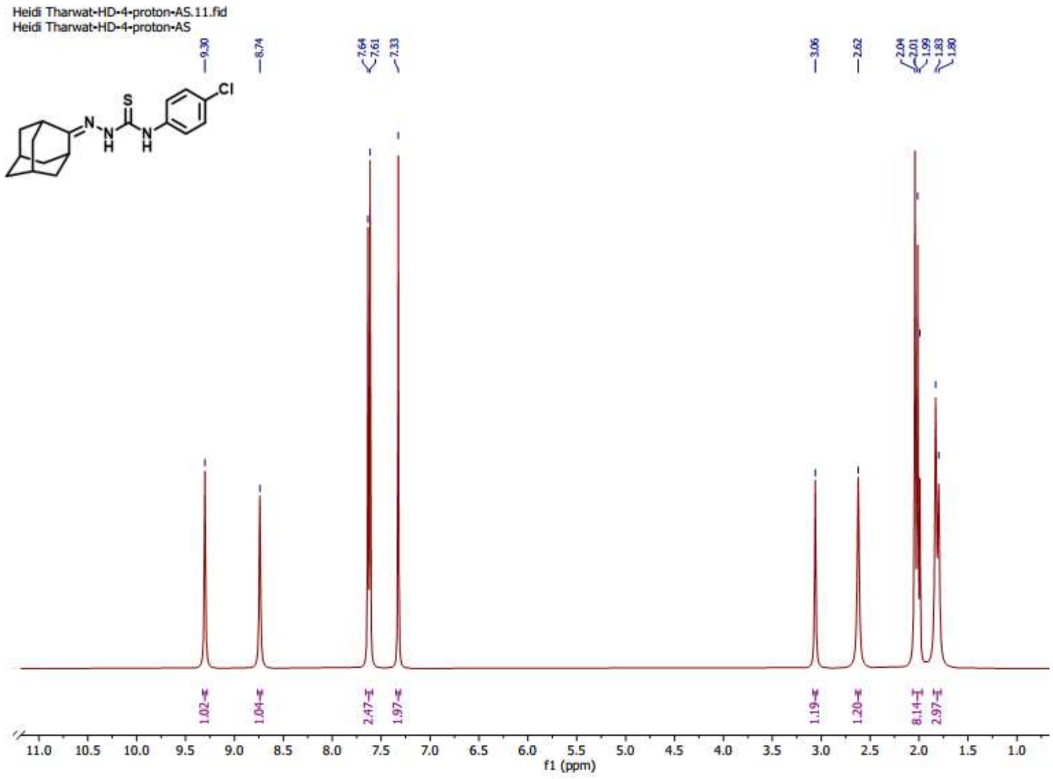

^{1}H NMR (400.20 MHz) of compound **5c**

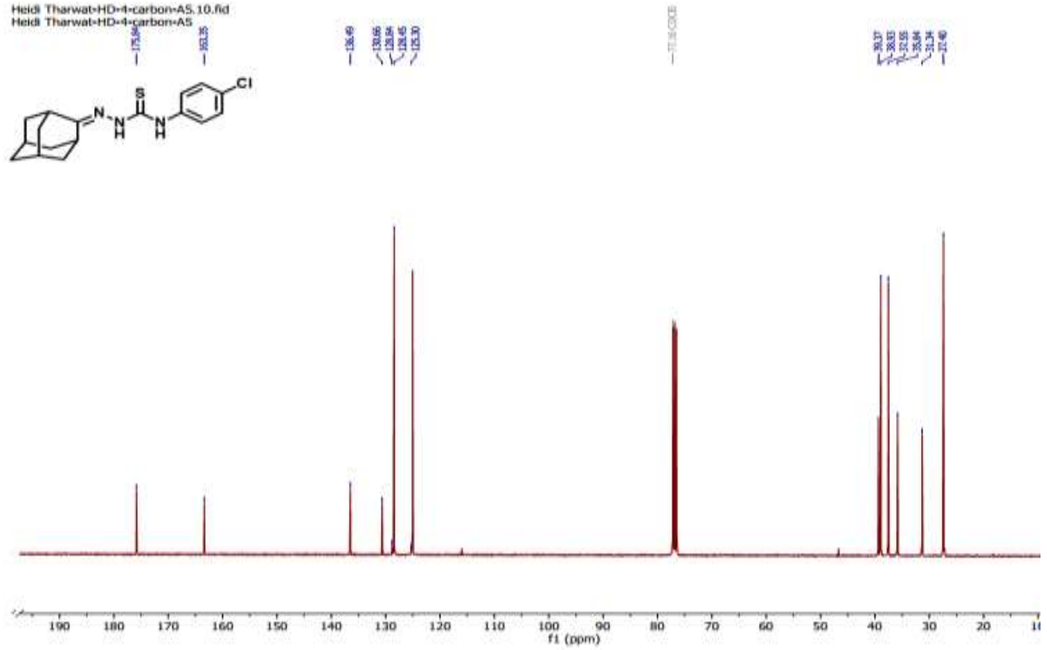

^{13}C NMR (100.63 MHz) of compound **5c**

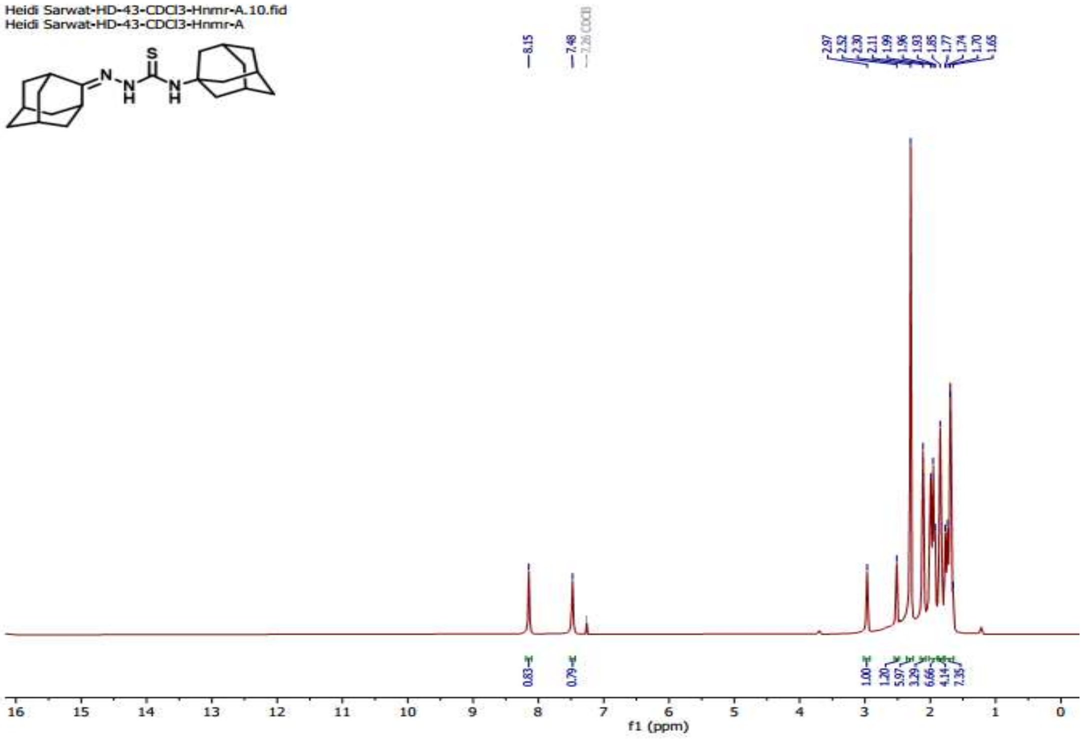

^{1}H NMR (400.20 MHz) of compound **5d**

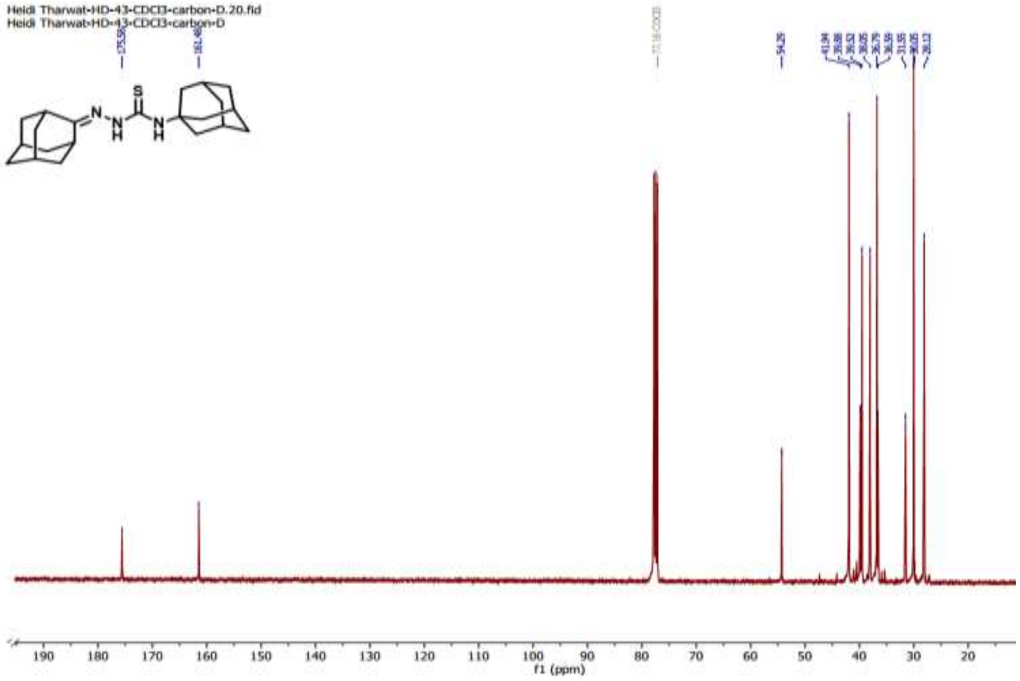

^{13}C NMR (100.63 MHz) of compound **5d**

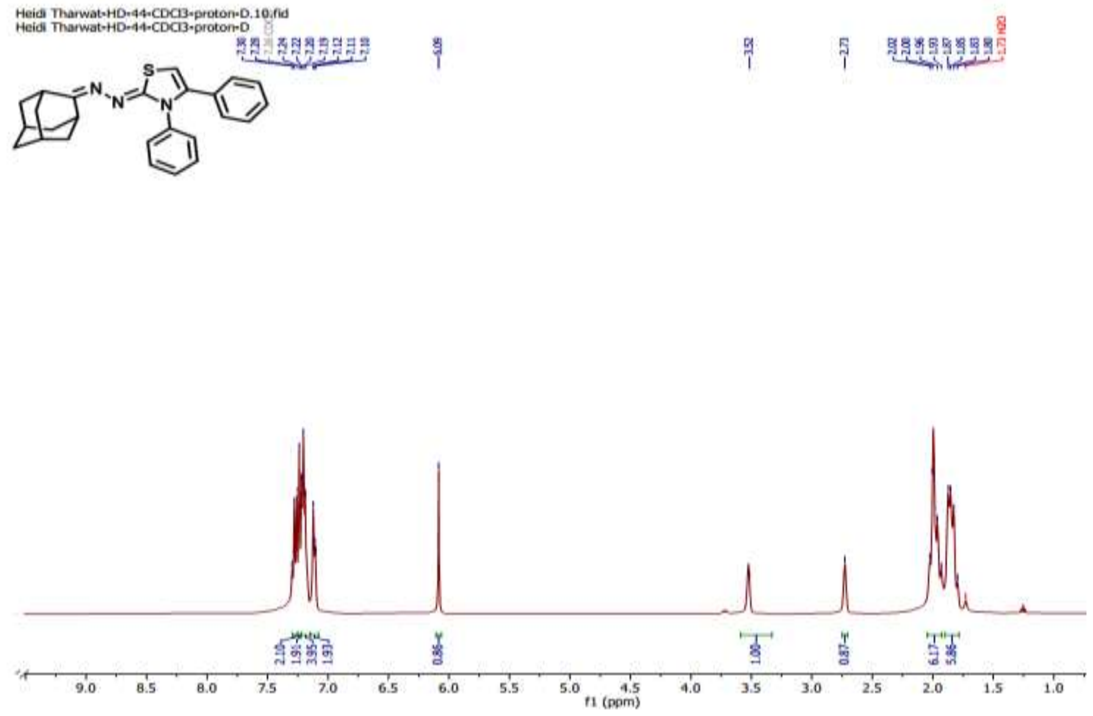

^{1}H NMR (400.20 MHz) of compound **7a**

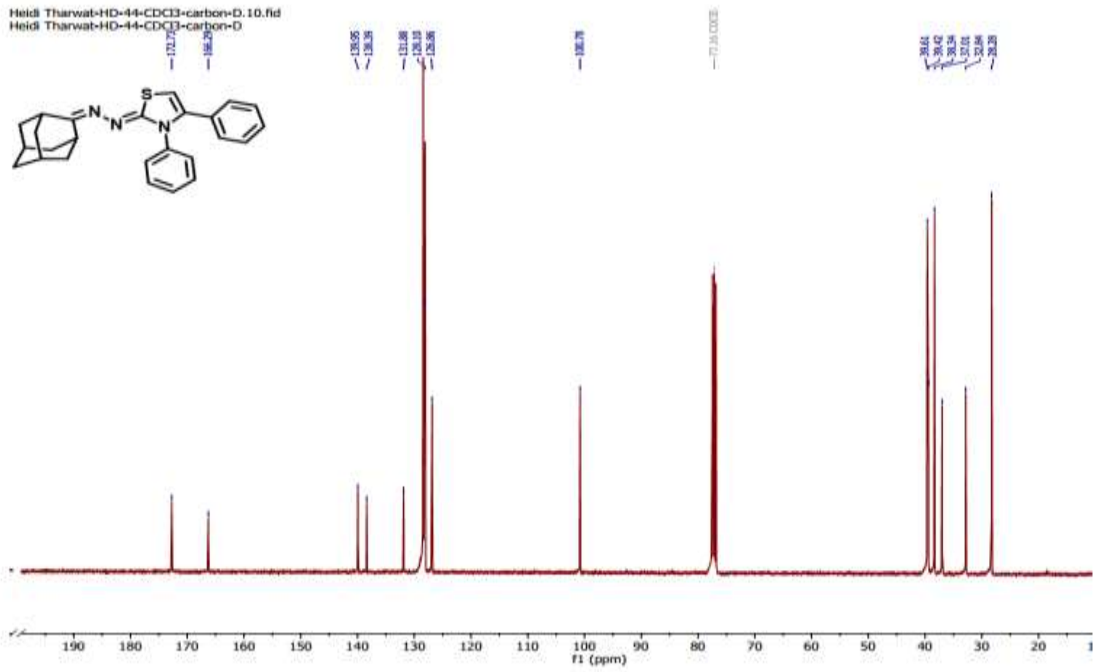

^{13}C NMR (100.63 MHz) of compound **7a**

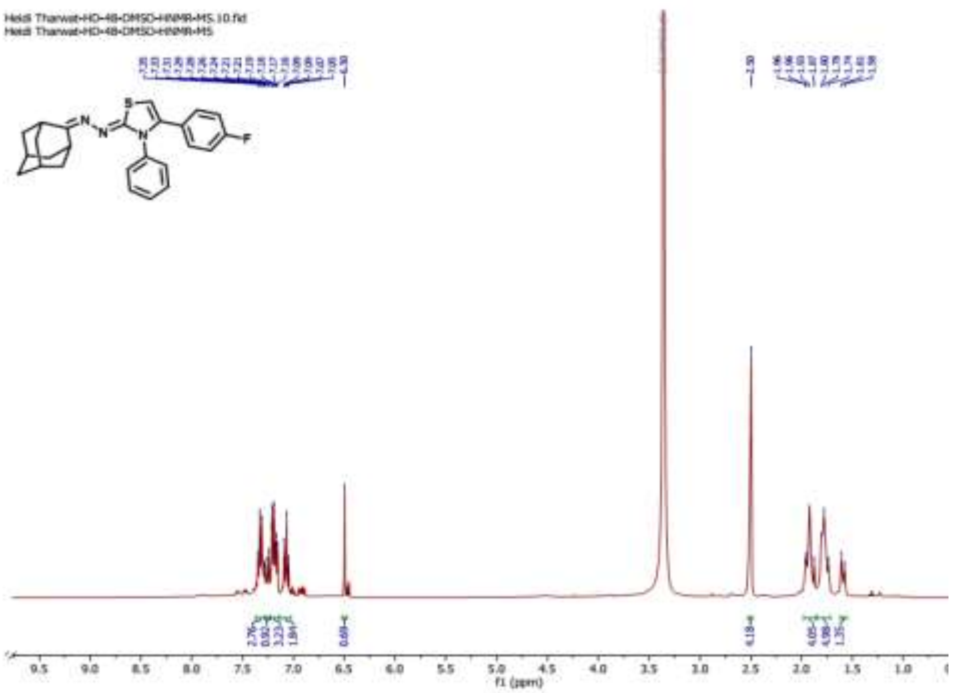

^{1}H NMR (400.20 MHz) of compound **7b**

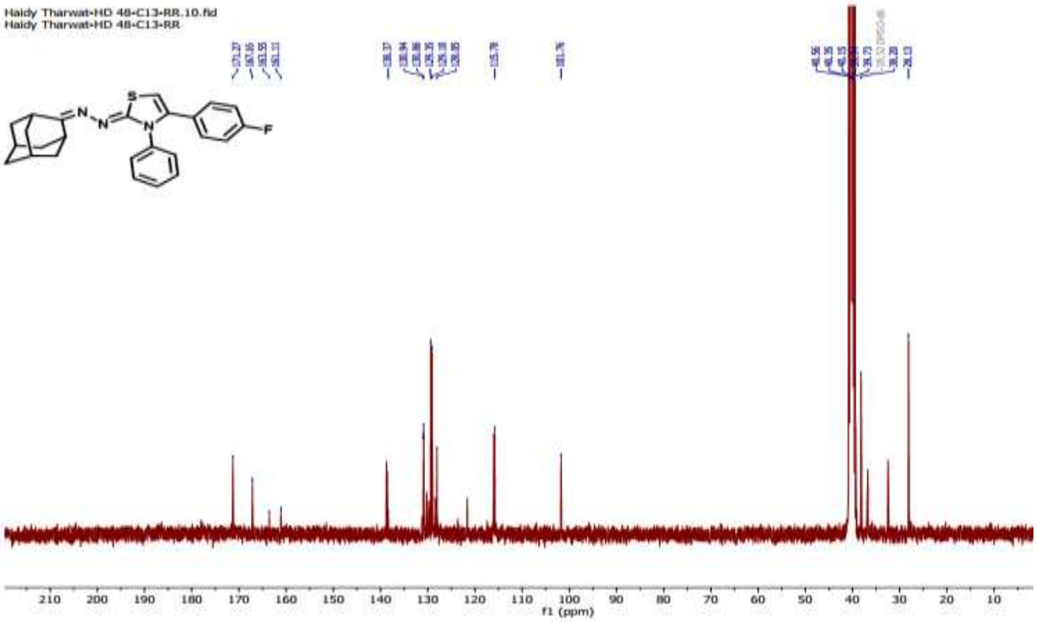

^{13}C NMR (100.63 MHz) of compound **7b**

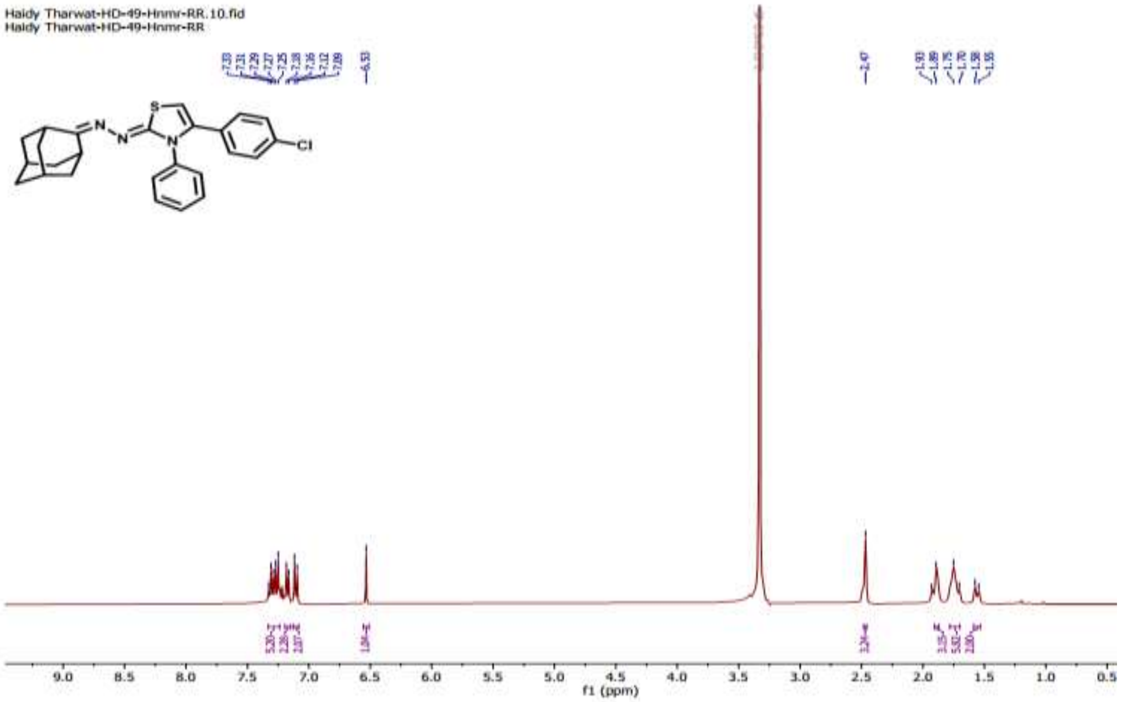

^{1}H NMR (400.20 MHz) of compound **7c**

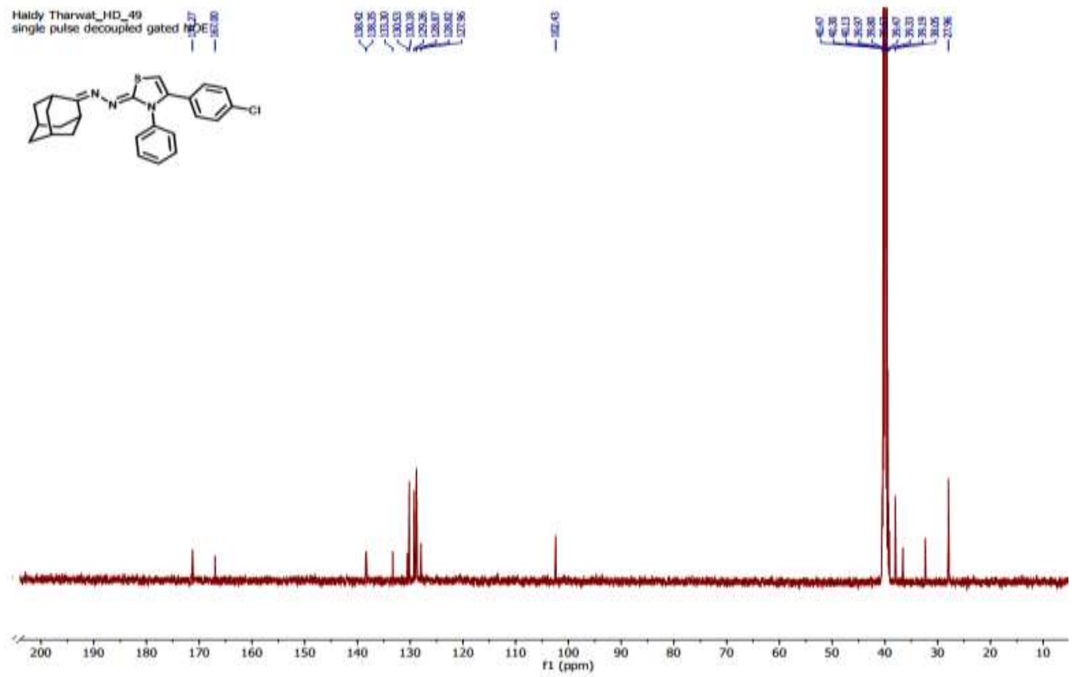

^{13}C NMR (100.63 MHz) of compound **7c**

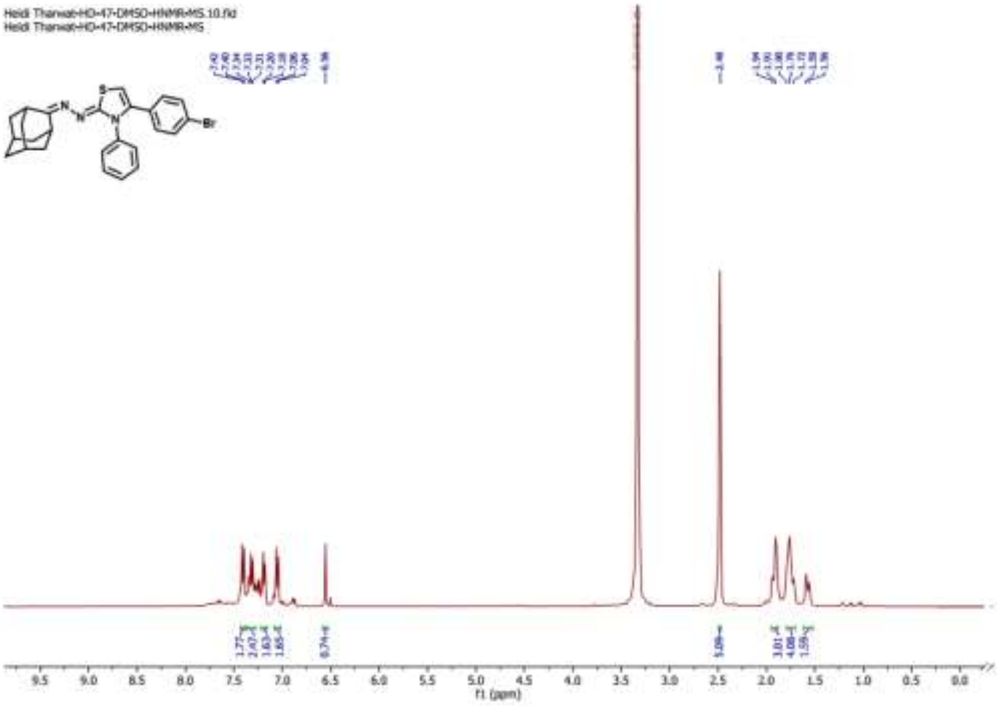

^{1}H NMR (400.20 MHz) of compound **7d**

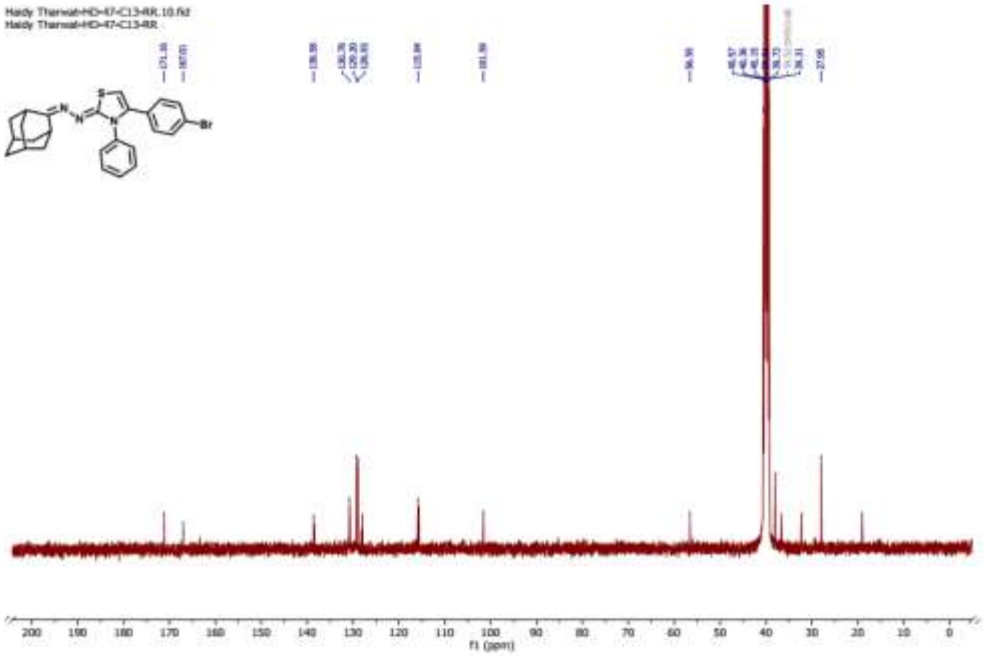

^{13}C NMR (100.63 MHz) of compound **7d**

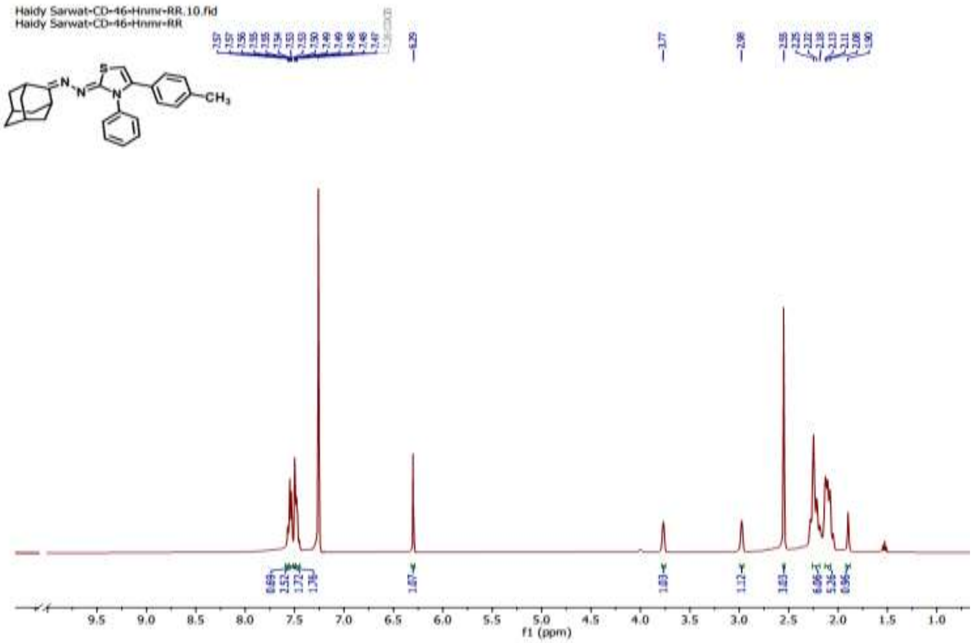

^{1}H NMR (400.20 MHz) of compound **7e**

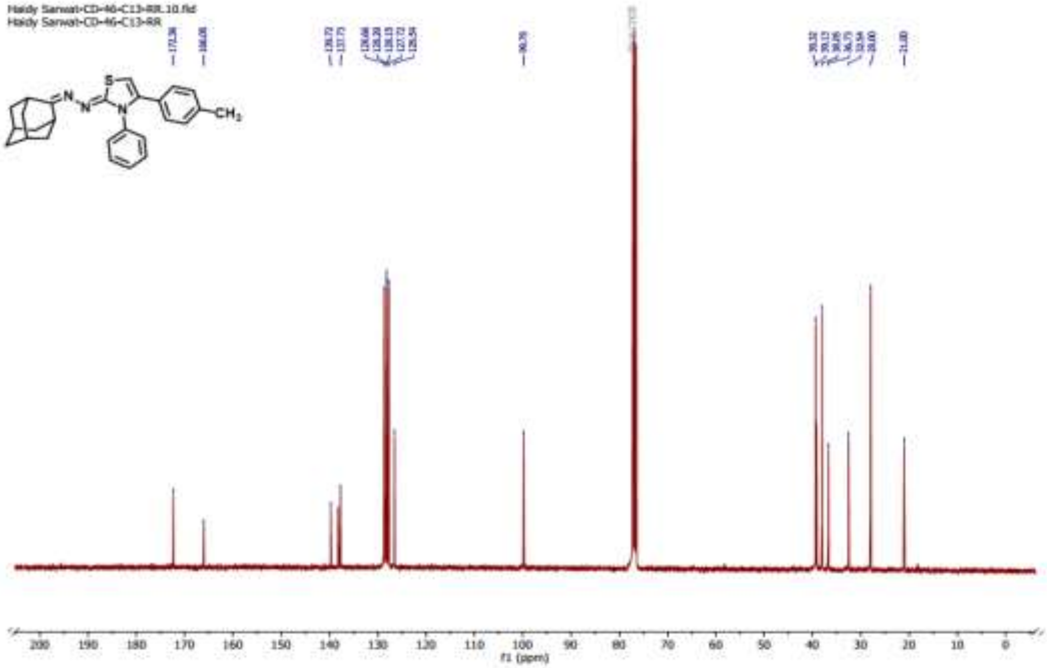

^{13}C NMR (100.63 MHz) of compound **7e**

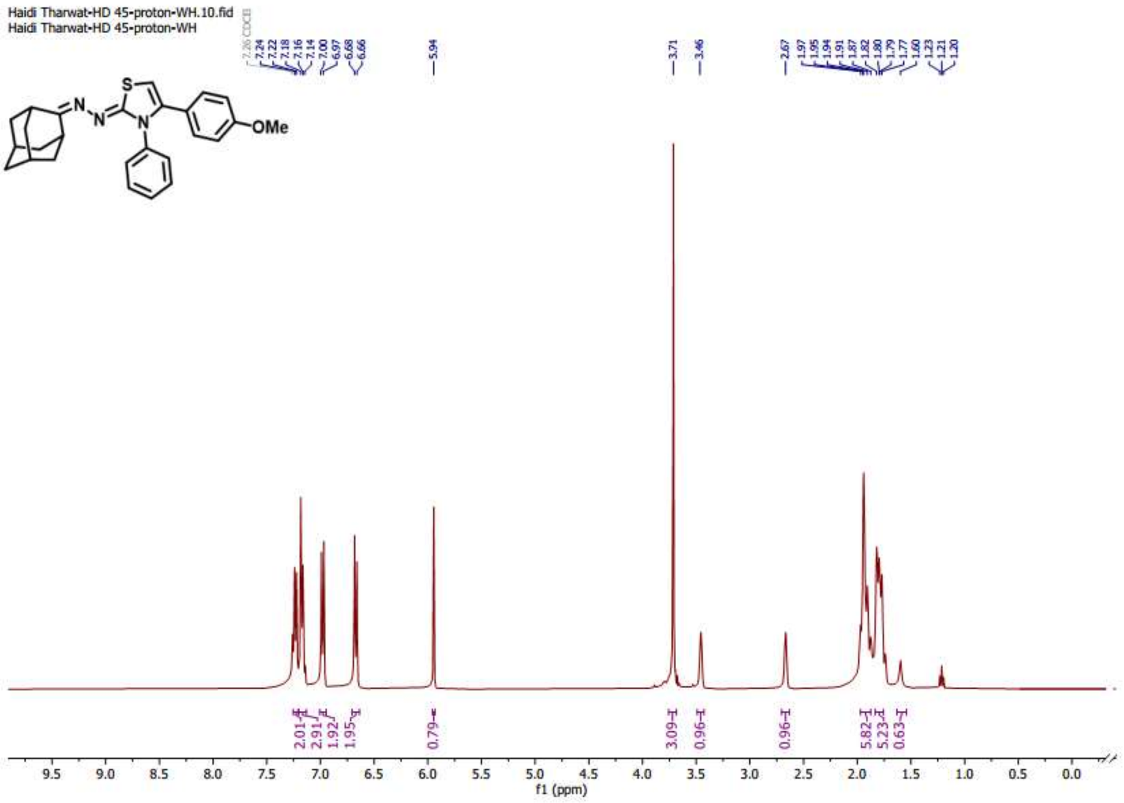

^{1}H NMR (400.20 MHz) of compound **7f**

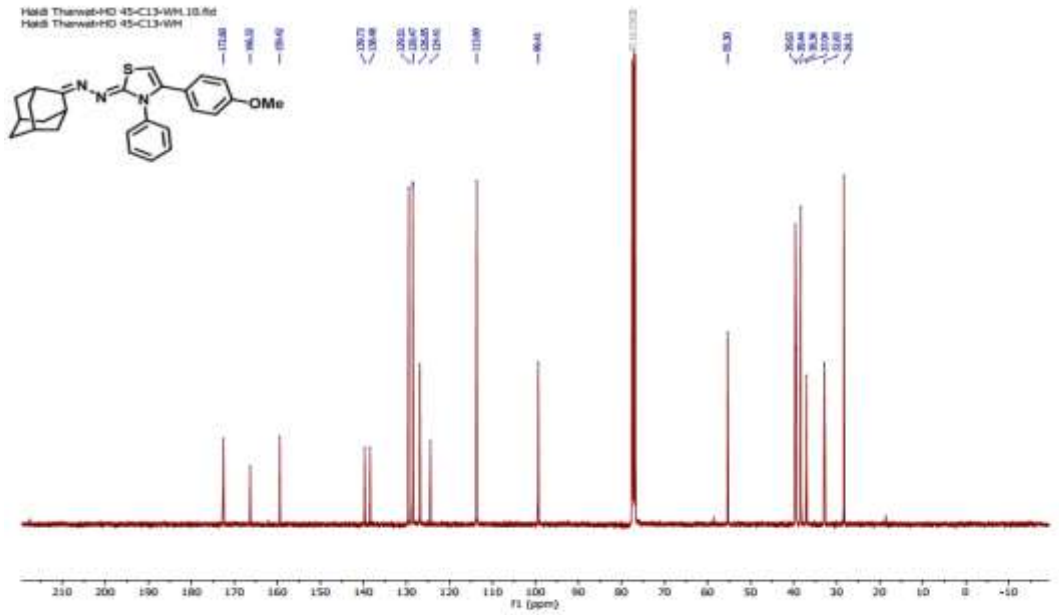

^{13}C NMR (100.63 MHz) of compound **7f**

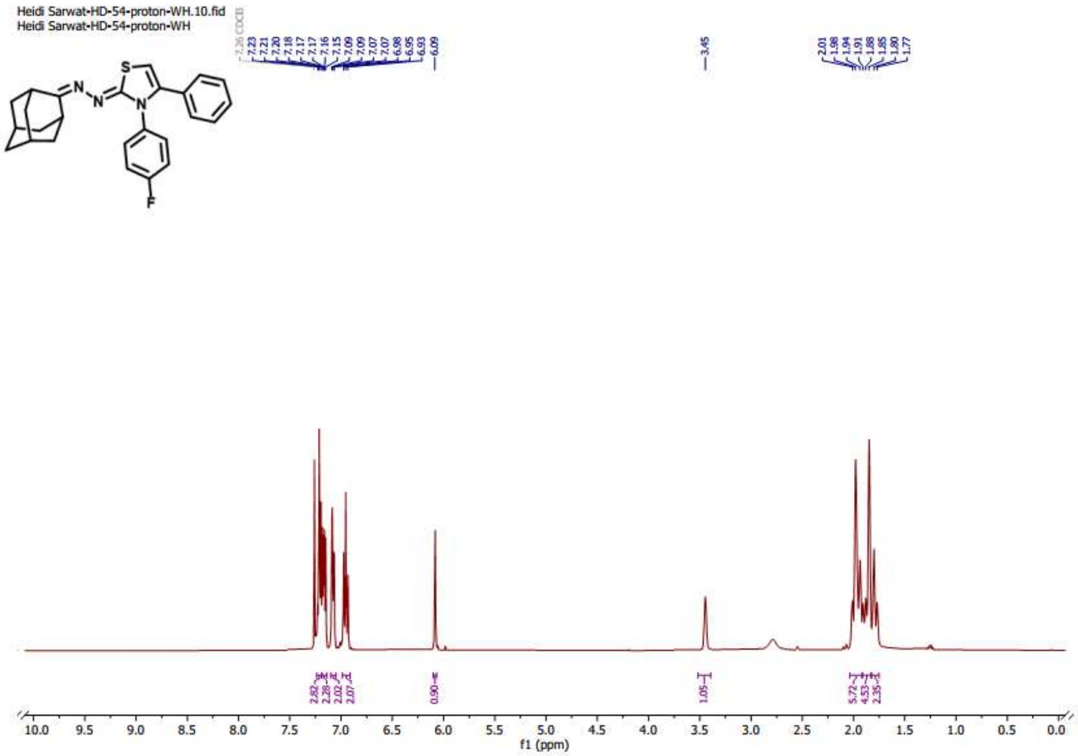

^{1}H NMR (400.20 MHz) of compound **7g**

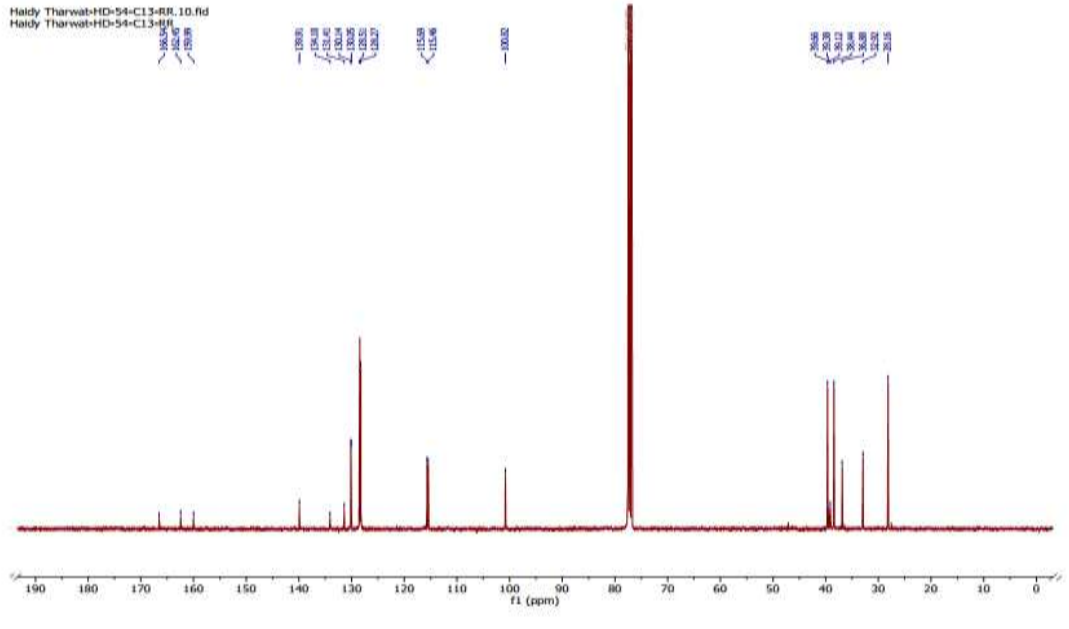

^{13}C NMR (100.63 MHz) of compound **7g**

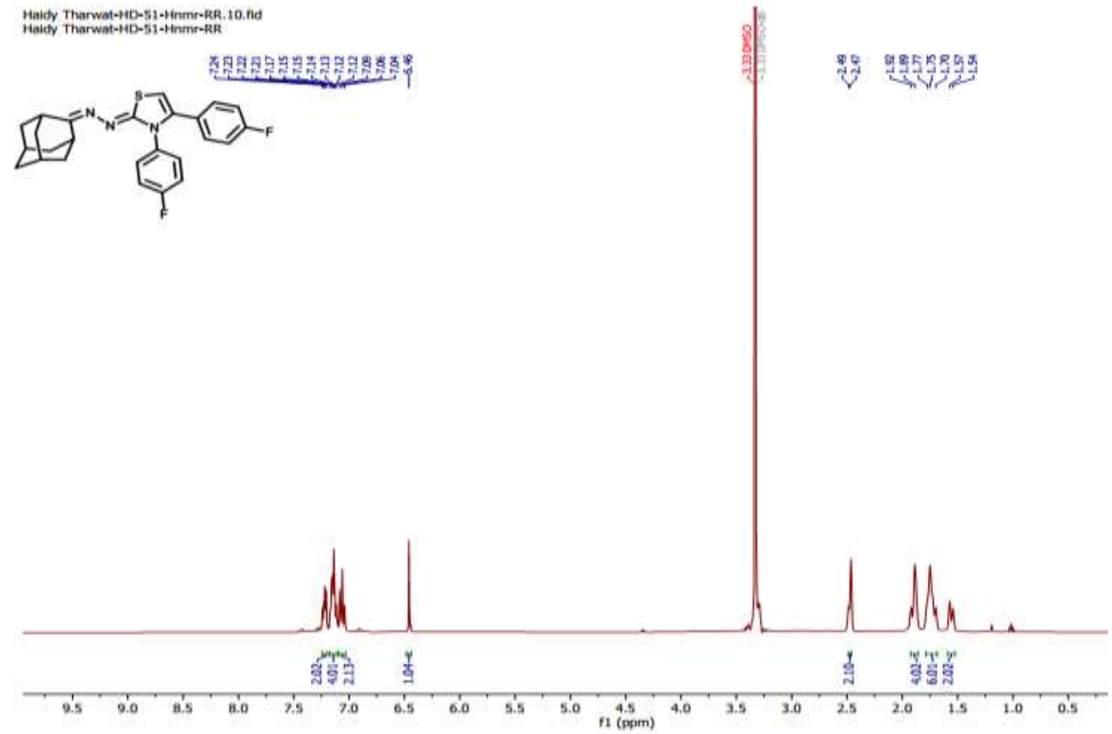

^{1}H NMR (400.20 MHz) of compound **7h**

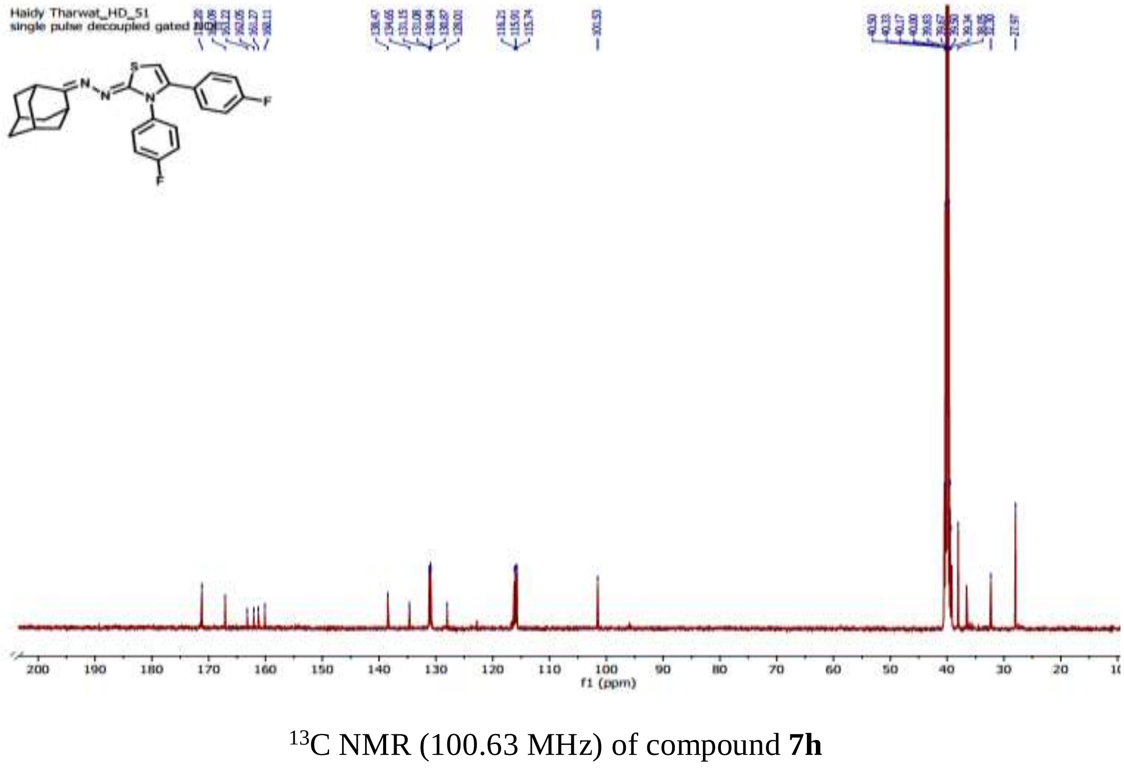

^{13}C NMR (100.63 MHz) of compound **7h**

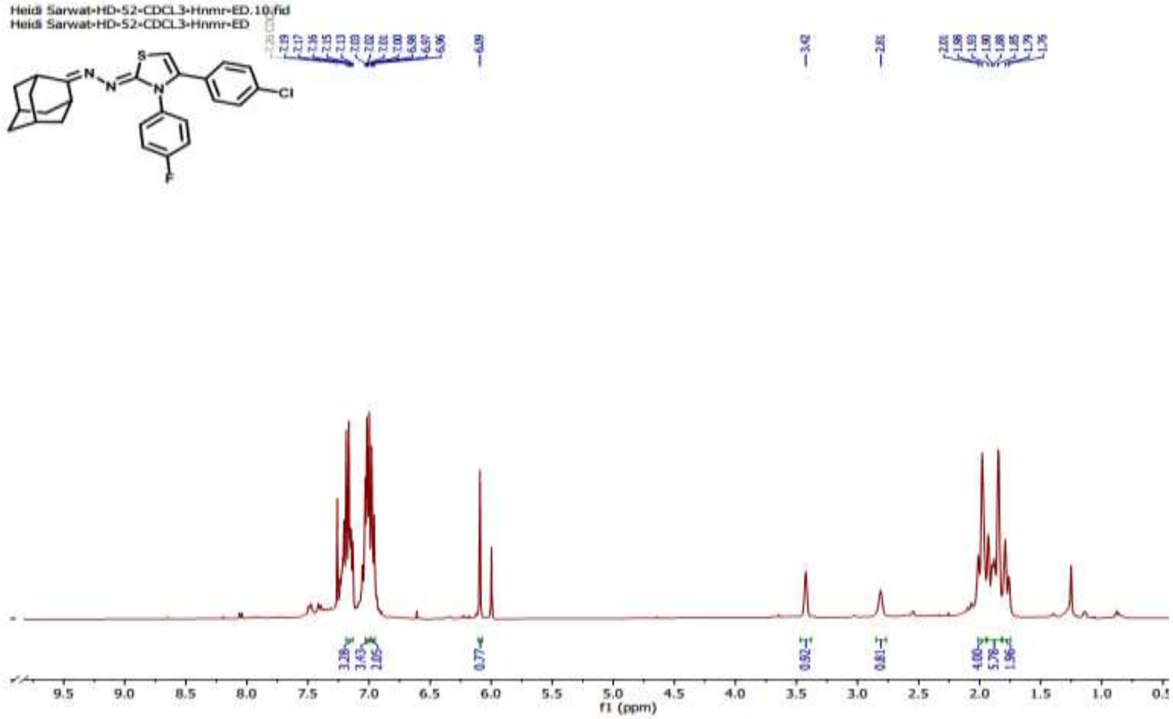

^{1}H NMR (400.20 MHz) of compound **7i**

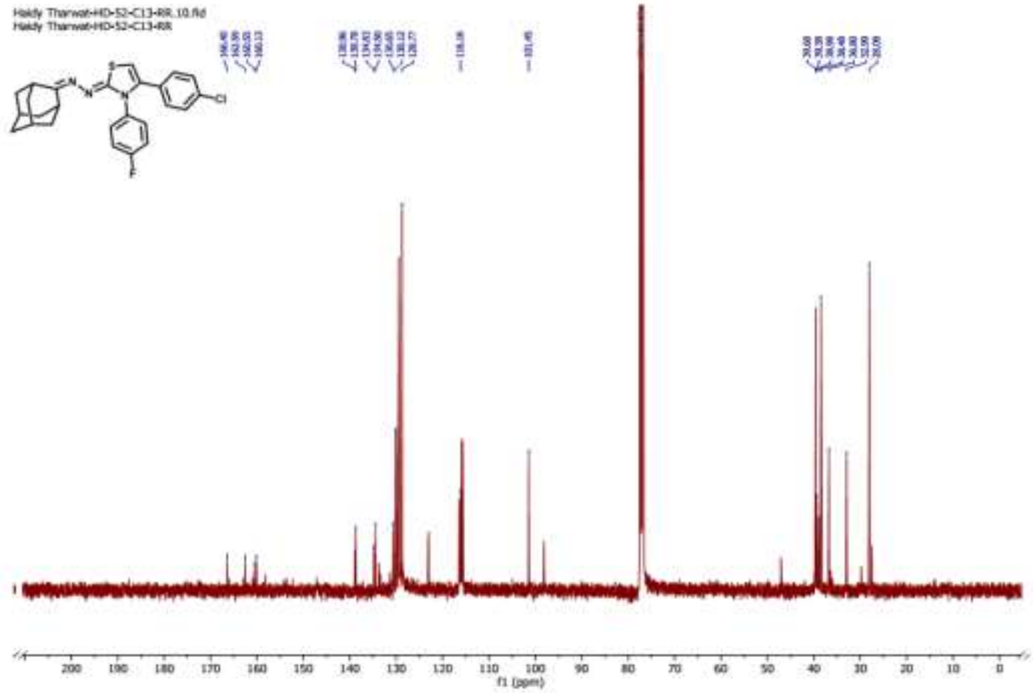

^{13}C NMR (100.63 MHz) of compound **7i**

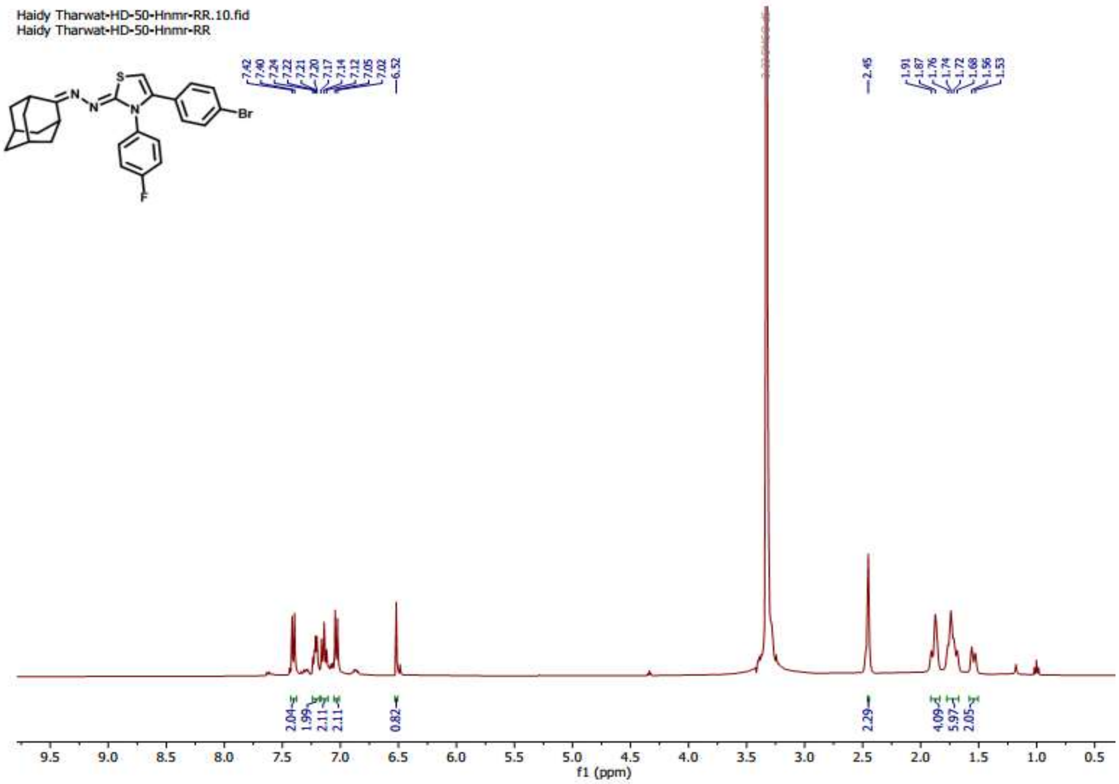

^{1}H NMR (400.20 MHz) of compound **7j**

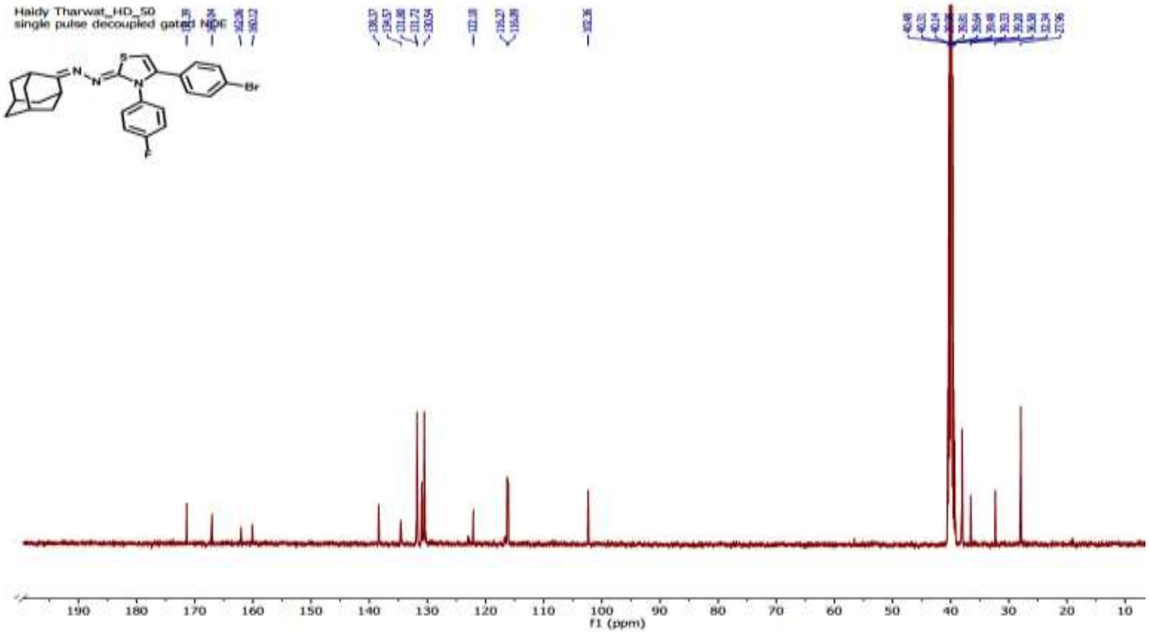

^{13}C NMR (100.63 MHz) of compound **7j**

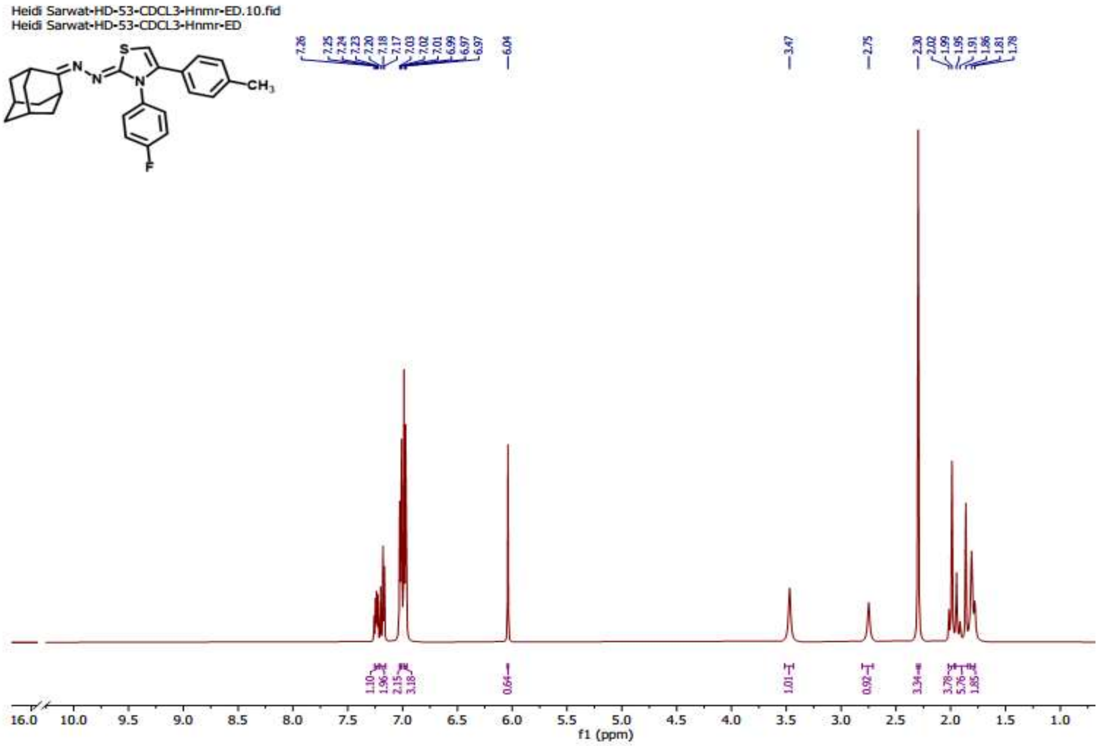

^{1}H NMR (400.20 MHz) of compound **7k**

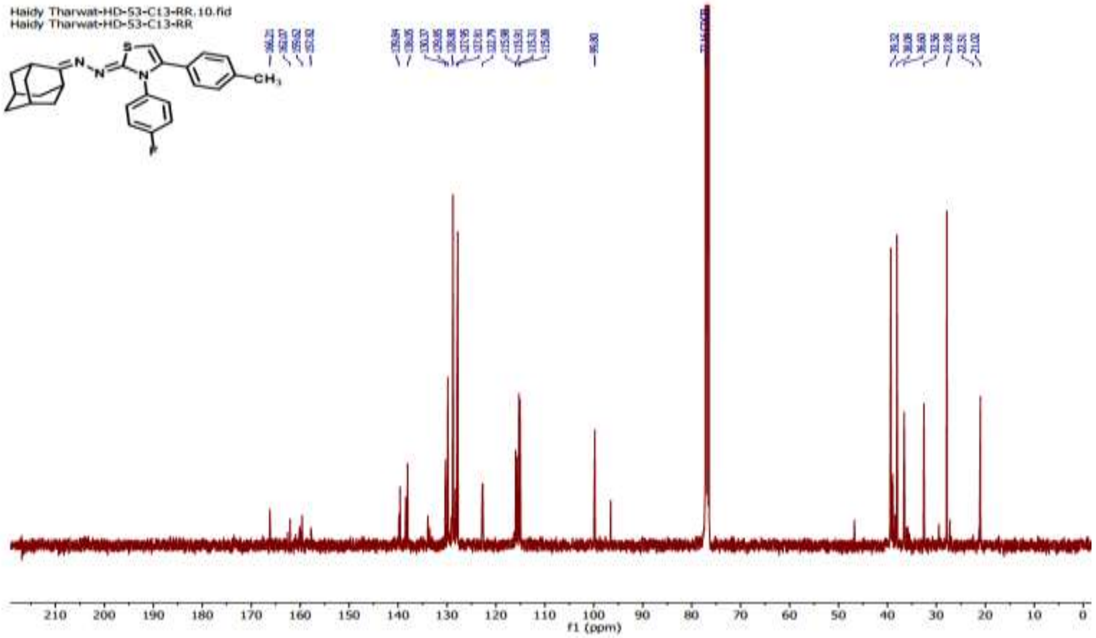

^{13}C NMR (100.63 MHz) of compound **7k**

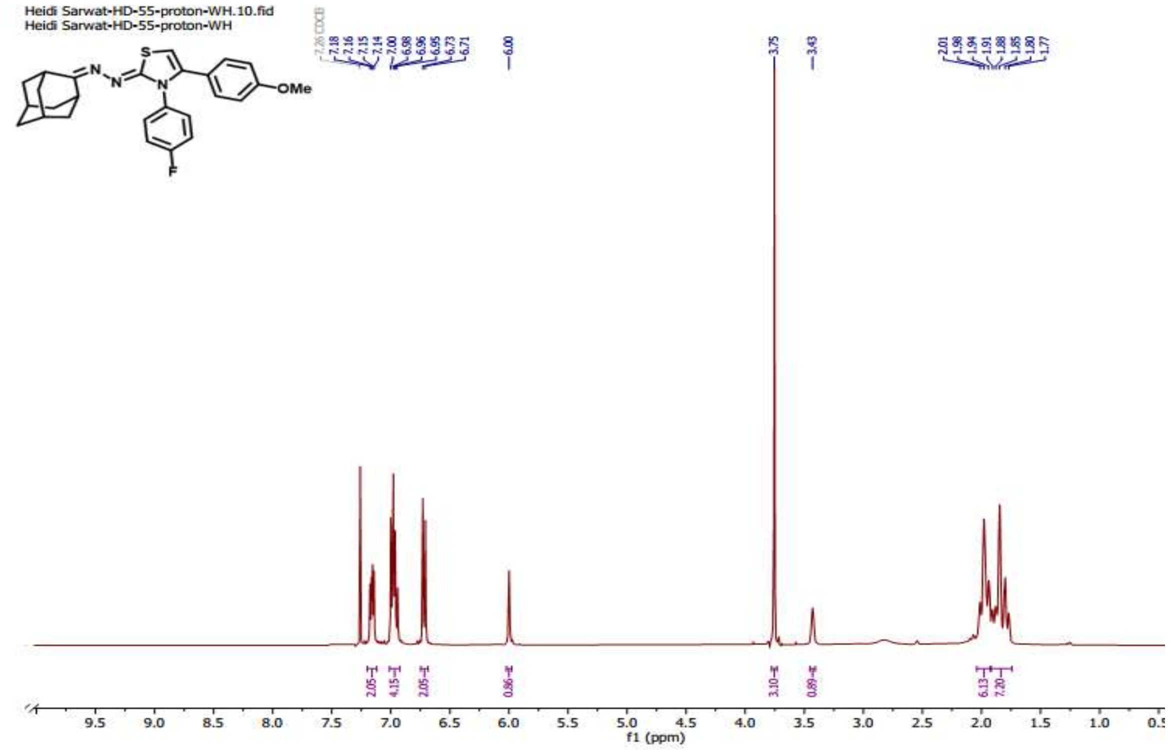

^{1}H NMR (400.20 MHz) of compound **7l**

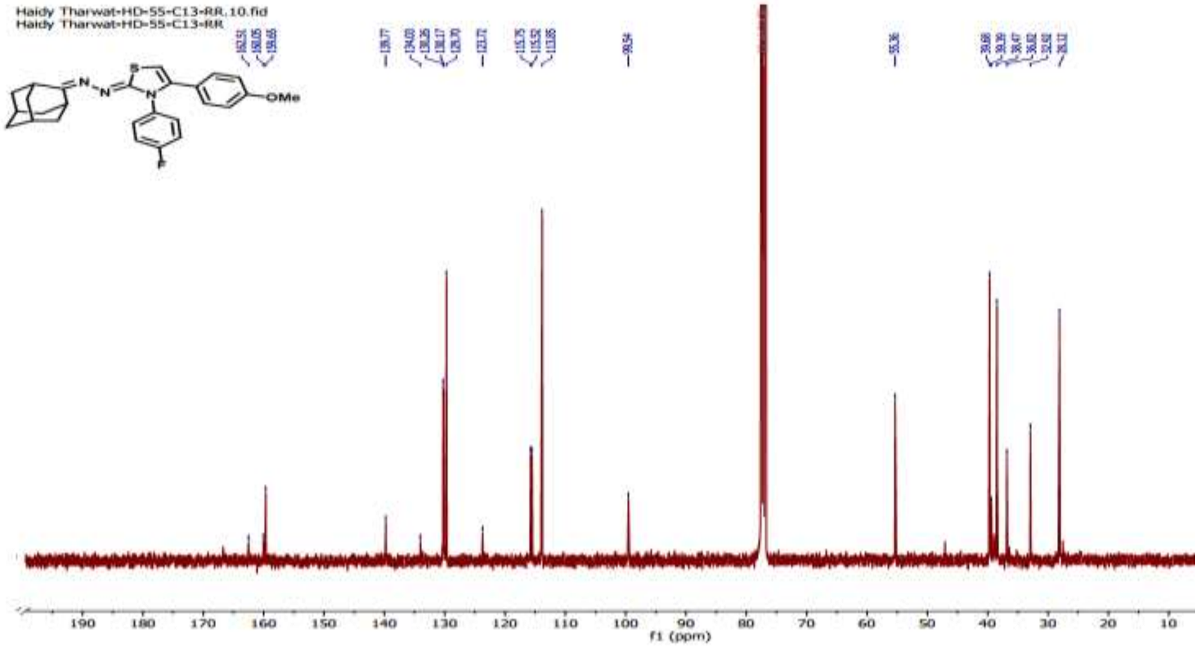

^{13}C NMR (100.63 MHz) of compound **7l**
